# Multi-constraints based deep learning model for automated segmentation and diagnosis of coronary artery disease in X-ray angiographic images

**DOI:** 10.7717/peerj-cs.993

**Published:** 2022-06-03

**Authors:** Mona Algarni, Abdulkader Al-Rezqi, Faisal Saeed, Abdullah Alsaeedi, Fahad Ghabban

**Affiliations:** 1 College of Computer Science and Engineering, Taibah University, Medina, Saudi Arabia; 2 Computer Science and Artificial Intelligence Department, University of Prince Mugrin, Medina, Saudi Arabia; 3 College of Medicine, King Saud bin Abdulaziz University, Jeddah, Saudi Arabia; 4 School of Computing and Digital Technology, University of Birmingham, Birmingham, United Kingdom

**Keywords:** X-ray angiography, Noise removal, Coronary artery disease, Segmentation, Classification, Attention-based nested U-Net, VGG-16

## Abstract

**Background:**

The detection of coronary artery disease (CAD) from the X-ray coronary angiography is a crucial process which is hindered by various issues such as presence of noise, insufficient contrast of the input images along with the uncertainties caused by the motion due to respiration and variation of angles of vessels.

**Methods:**

In this article, an Automated Segmentation and Diagnosis of Coronary Artery Disease (ASCARIS) model is proposed in order to overcome the prevailing challenges in detection of CAD from the X-ray images. Initially, the preprocessing of the input images was carried out by using the modified wiener filter for the removal of both internal and external noise pixels from the images. Then, the enhancement of contrast was carried out by utilizing the optimized maximum principal curvature to preserve the edge information thereby contributing to increasing the segmentation accuracy. Further, the binarization of enhanced images was executed by the means of OTSU thresholding. The segmentation of coronary arteries was performed by implementing the Attention-based Nested U-Net, in which the attention estimator was incorporated to overcome the difficulties caused by intersections and overlapped arteries. The increased segmentation accuracy was achieved by performing angle estimation. Finally, the VGG-16 based architecture was implemented to extract threefold features from the segmented image to perform classification of X-ray images into normal and abnormal classes.

**Results:**

The experimentation of the proposed ASCARIS model was carried out in the MATLAB R2020a simulation tool and the evaluation of the proposed model was compared with several existing approaches in terms of accuracy, sensitivity, specificity, revised contrast to noise ratio, mean square error, dice coefficient, Jaccard similarity, Hausdorff distance, Peak signal-to-noise ratio (PSNR), segmentation accuracy and ROC curve.

**Discussion:**

The results obtained conclude that the proposed model outperforms the existing approaches in all the evaluation metrics thereby achieving optimized classification of CAD. The proposed method removes the large number of background artifacts and obtains a better vascular structure.

## Introduction

**Figure 1:**
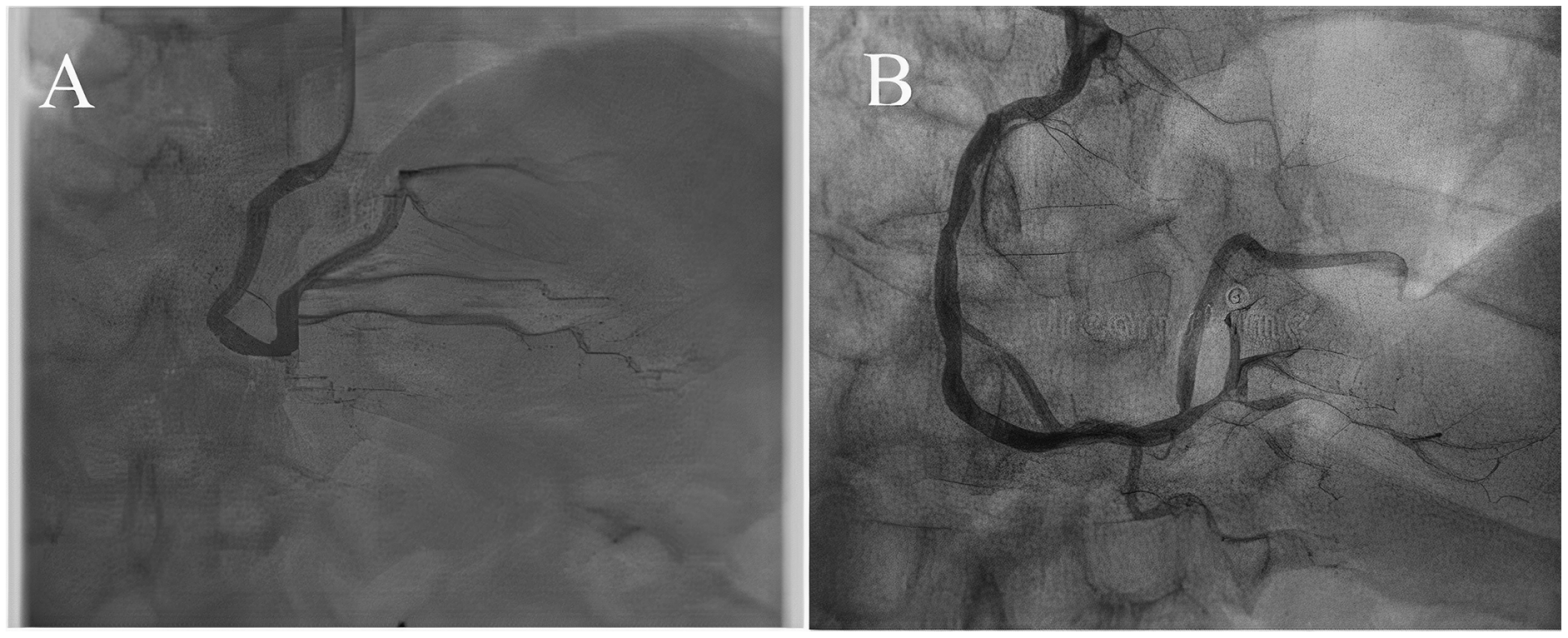
(A) and (B) X-ray angiography with noise and artefacts.

**Figure 2:**
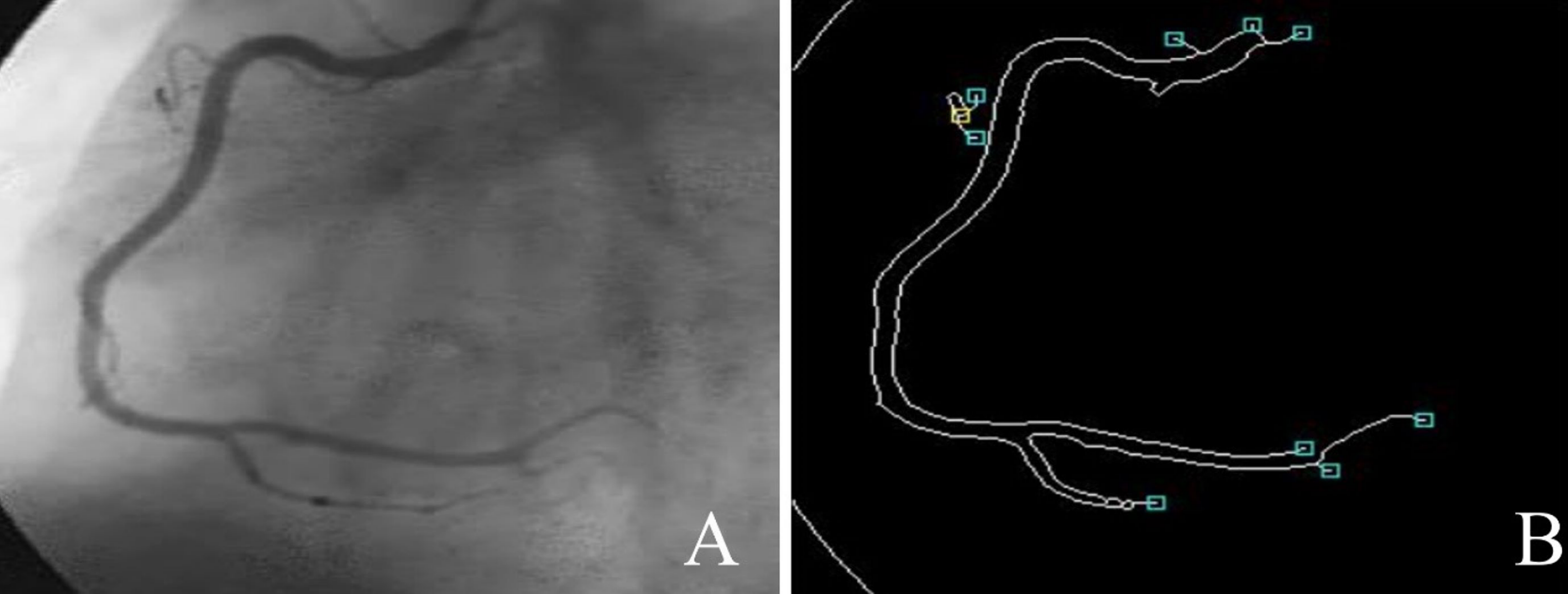
(A) X-ray angiography, (B) Segmented image with stenosis.

Coronary artery disease (CAD) is the most significant type of heart disease. CAD occurs when atherosclerotic plaque builds up in the coronary artery wall (Hong et al., 2019). Figure 1 depicts an X-ray angiography comprised of noise and artifacts, which affect the proper detection of CAD. Recently, X-ray coronary angiography, coronary computed tomography, and coronary heart disease dataset images have proven to be effective diagnostic tools for CAD detection (Kong et al., 2020; Wang et al., 2020b). In these images, the core information of CAD can be analyzed and extracted for cardiovascular disease prediction; this information includes flow velocity and vessel diameter (Chow et al., 2021). Analyzing CAD may be hindered due to low contrast, small vessels, structural interference, breathing motions, and artifacts/noise (Hajhosseiny et al., 2021). Because of these artifacts, blood vessel extraction from X-ray images must be effective. The semantic segmentation of coronary vessels is extremely important in the clinical process of cardiology analysis (Zou et al., 2020). Both left coronary artery (LCA) and left anterior descending (LAD) artery segmentation with stenosis are tedious and time-consuming in the existing methods, as depicted in Fig. 2 (Zhang et al., 2020; Bahaaeldin et al., 2019; Bom et al., 2019).

In the related literature, the challenge of automatic segmentation and classification of the coronary artery has been widely investigated by various methods (Koo et al., 2020; Zhou et al., 2021). Existing methods have applied manual vessel extraction for X-ray coronary angiography images (Bratt et al., 2019). However, these methods are greatly affected by human errors, lack of accuracy, and low consistency (Franks, Plein & Chiribiri, 2021). Hence, automatic coronary artery extraction methods are being investigated to minimize inaccuracy in CAD prediction (Orlenko et al., 2020).

Many machine learning methods, such as linear regression and support vector machines (SVM), have been utilized to detect CAD (Zreik et al., 2020; Abdar et al., 2019b). However, examining X-ray images manually consumes more processing power, and it is also tedious. It leads to inter- and intra-observer errors (Li et al., 2021). Many deep algorithms have been proposed, such as the convolutional neural network (CNN) and recurrent neural network (RNN) (Shen et al., 2019; Wolterink et al., 2019). In X-ray angiography images, coronary artery extraction/segmentation is a significant process that plays a vital role in CAD prediction. Many algorithms, such as the Hessian matrix method, mathematical morphology, and the minimal cost path technique, have been developed (Rim et al., 2021).

The main aim of this research is to enhance the accuracy of the coronary artery disease diagnosis system by developing novel algorithms for the segmentation and detection processes. The diagnosis system involves three processes: preprocessing, segmentation, and classification *via* the extracted features from each artery. Thus, this article investigates the diagnosis of coronary artery in X-ray angiography images by extracting the blood vessel effectively from X-ray image samples and facilitating the preprocessing tasks to classify the images as low quality, high artifacts, *etc*.

The diagnosis of CAD using X-ray angiograms is an emerging medical field that predicts heart-related diseases of patients. The use of X-ray angiograms is simple and low cost, but it does not produce quality images initially. The artifacts in X-ray images are low contrast with high distortion in the foreground and background pixels. These issues with X-ray images affect the performance of segmentation and classification metrics, such as peak signal-to-noise ratio (PSNR), segmentation accuracy, and classification accuracy. Existing methods in coronary artery segmentation and classification are not efficient in terms of addressing these issues. The most significant research problems undertaken in this article were as follows:

- Lack of preprocessing: In CAD diagnosis, it is important to analyze the quality of coronary artery images, and it must meet the constraint of coronary arteries to be structured properly. This must be thoroughly studied before the segmentation and prediction processes.
- Static thresholding: This problem is fixed and unsolved in coronary artery segmentation; however, coronary artery vessels are different in length, orientation, and diameter according to the patients. A static threshold is not suitable for the accurate segmentation of the coronary artery, and it causes low accuracy in micro heart disease prediction (*e.g*., stenosis).
- Computational complexity: Existing works use heavyweight approaches, such as CNN, RNN, and matrix models, which consume high memory storage and computation power for the algorithms and also take up more processing time for both segmentation and classification.
- The proposed methods are not suitable for small blood vessels. CAD can be possible in both large and small types of blood vessels. Furthermore, dynamic pixel selection deviates for all types of images, and the effects of measuring the diameters for small blood vessels are unstable.
- A very limited number of features, such as Laplacian zero crossing, gradient magnitude, gradient direction, edge pixel value, inside pixel value, and outside pixel value, are not sufficient for effective segmentation.
- The diameter value is fixed but different for various types of patients, and it results in poor segmentation performance.

Therefore, in this article, we proposed a novel model, automated segmentation coronary artery disease (ASCARIS), to diagnose the coronary artery using the deep learning method. The contributions of this article were as follows:

- First, we applied preprocessing processes, such as noise removal and contrast enhancement. We applied a modified Wiener filter to remove noise from the images. This method was used to recover the original images from the distorted images. For contrast enhancement, we applied Otsu thresholding, which enhanced the contrast of the images to increase image quality.
- Second, we performed coronary artery vessel extraction for this research by using an attention-based nested U-net, which segmented all the arteries in the image. Artery angle-based segmentation was proposed to improve segmentation and classification accuracy.
- Third, we used the VGG16 method for feature extraction and classification. To detect the coronary artery, we extracted color, shape, and texture features from the segmented result. After extracting the features, an attention estimator was used to calculate the weight values of the features’ importance based on the results. Then, we classified the results into two classes: normal and abnormal.

The performance of the proposed approach was analyzed by various evaluation metrics, such as accuracy, sensitivity, specificity, ROC curve, revised contrast-to-noise ratio, mean square error, dice coefficient, Jaccard similarity, Hausdorff distance, PSNR, and segmentation accuracy. It was compared with previous methods to measure the performance of the ASCARIS model.

The rest of this article is organized as follows: “Related Studies” explains related works on the diagnosis of the coronary artery using CT images. “Materials and Methods” describes the problems that exist in the previous works. “Results” explains the proposed ASCARIS model in detail, including the pseudocode, algorithm, and mathematical representation. “Discussion” summarizes the simulation results of the proposed ASCARIS model, which are compared with the existing methods using various performance metrics. “Conclusion” concludes the result and performance of the proposed ASCARIS model and also includes the future work of this research.

## Related Studies

In this section, a survey on the segmentation of the coronary artery and the detection of CAD using X-ray angiography was conducted. From this, the research gap in designing a model to achieve improved accuracy in the detection of CAD was formulated.

Coronary artery segmentation has been presented in X-ray angiography videos (Wang et al., 2020a). The spatial and temporal information extraction method was applied to automatically segment the artery in a video. From the video, the numbers of frames were extracted, then the spatial and temporal were applied to extract the coronary artery. The spatial and temporal information was extracted using integrated deep learning algorithms, such as the 3D convolutional neural network and 2D convolution layer. The source image and ground truth image were compared with several existing algorithms, such as U-net, AG-net, CE-net, 3D-2D net *N* = 1, and 3D-2D net *N* = 2. Finally, the proposed 3D-2D net methods proved better accuracy. However, the spatial information extracted from X-ray images was computationally very expensive. Extraction with 3D CNN and 2D net was also time-consuming. A new deep learning-based segmentation method has been proposed for X-ray angiograms (Fan et al., 2018). In this method, a multichannel-based fully convolutional neural network (FCNN) was applied for accurate segmentation *via* distinguishing the real blood vessel structure. Next, hierarchical feature extraction was applied to further segment the coronary artery. In this step, the spatial relationship between the background structure and vessel was predicted. Finally, dense matching was applied between the mask and live image for accurate initial alignment. However, this method required a live image to extract the blood vessel structure, which is not always obtainable in real-time. The angiography image matching method has also been proposed, which follows the hierarchical dense matching framework (Fan et al., 2019). This was a CNN-based feature descriptor that designed correlation maps for the appropriate multi-feature maps generation. The proposed method used SIFT, a deep matching descriptor for generating feature maps over a number of patches. Dense matching was performed with three kinds of patches: source patch, similar patch, and dissimilar patch. The main limitation was that background subtraction was necessary to improve the quality of CAD images. The lack of preprocessing operation and consideration of the max pool layer in CNN did not enhance accuracy. One of the significant blood vessels in the heart was segmented, which was located in the left anterior (Jo et al., 2019). However, the segmentation of major vessels from a multiple blood vessel set was a very important problem due to the presence of complex and small vessels, background structure, low contrast ratio, and non-uniform illumination. To deal with those, selective feature mapping was proposed in our work. Two phases were involved in a previous study: candidate area segmentation and segmentation. The acquired image was preprocessed, segmented, and finally, post-processed in segmentation. After overlapping the ground truth image with the angiography image, an expert was required to identify and correct errors in the candidate area. The manual segmentation method did not produce higher accuracy in a shorter time.

A set of image sequences (*i.e*., X-ray) has been considered for cardiovascular enhancement (Song et al., 2020). For that, the spatio-temporal constrained online layer separation (STOLS) method was presented to achieve X-ray angiogram (XRA) image sequences. This method integrated the consistency of motion structured and temporal constrained online robust principal component analysis used to improve the contrastness of vascular images. However, this method did not increase accuracy in real time, since it was not sufficient for predicting CAD in the XRA sequence, and it did not show new and small vascular segments between sequences of angiograms. The inter/intra-frame constrained vascular segmentation model for segmenting the coronary X-ray angiography image sequence was used in Song et al. (2019). For that model, morphological filtering was used to remove the motion structure from the original set of image sequences. Then, inter-frame constrained principal component analysis (RPCA) was performed to eliminate the quasi-static structure in the set of X-ray images to smooth over the final extracted vascular sequence. Finally, multi-feature fusion was applied to improve the contrast level of the vascular images, and thus, the final vascular segmentation for thresholding. However, RPCA produced high dimensionality in feature extraction, which increased the overhead in vessel segmentation. Furthermore, the random scheme for multi-feature fusion did not effectively support segmentation. Image-based coronary artery segmentation was presented for accurate CAD classification in Wan et al. (2018). In that study, the Hessian matrix and statistical region merging were used, which could easily extract the complex vessel structure and vessel delineation. The steps in the operation were noise reduction and background homogenization, Hessian-based vascular structure enhancement (structure preservation), and statistical vessel segmentation (region growing-based pixel selection and sorting and post-processing). There were several problems in this study: (1) the non-vascular structure was not accurately segmented by this method because the vessels intersected and were close to the vascular branch; (2) the tubular-based structure induced artifacts but were improved by the Hessian method, causing a high false positive rate; and (3) thin vessels were imprecisely identified. Therefore, the overall study did not focus on vessel pathology.

Morphology information was quantized in the set of X-ray angiography images in Zhao et al. (2019). However, morphological information was significant for coronary artery disease detection. For that reason, a directed graph construction was presented in the automatic process of morphology construction. This method consisted of three steps: undirected graph construction, edge direction estimation, and directed graph construction. First, an undirected graph was constructed for the primary topology representation in the vessel tree. Then, the vessel centerline was segmented, splatted, and reassembled into the edges of the undirected graph. Second, every edge was allocated to a direction using an iteration function in which the graph representation and geometric features of the vessel were considered as the constraints. The limitation of this study was that the graph construction time was very high, which did not suit real-time disease diagnosis applications.

To detect the disease, coronary artery segmentation was performed using a deep learning algorithm in Xiao, Li & Jiang (2020). The U-net algorithm was proposed for deep coronary artery segmentation to predict the risk of disease. The local features were extracted from the segmented image for risk prediction. This method used quasi-Newton gradient descent to calculate and update weight parameters. The simulation result showed that this model achieved high performance in terms of dice coefficient compared with other methods. However, this study used raw images for segmentation, which led to poor segmentation due to the presence of noise and other artifacts, thus reducing segmentation accuracy.

Coronary artery disease was detected by extracting the blood vessel centerline using CT coronary angiography images in Sheng et al. (2019). First, the coronary artery was refined, resulting in a rough centerline and eliminating wrong branches, loops, and gaps. The distance transformation method was proposed to extract the centerline. The Euclidean distance was calculated between the points with respect to boundaries. Then, it automatically selected the seed points. This method used a tracking method to track the centerline position and direction. If the distance between the center points was large, then it was considered as the normal centerline.

Diagnosing coronary artery disease (CAD) using an ensemble clinical decision support system was presented in Abdar et al. (2019a). A nested ensemble support vector classification was proposed for the diagnosis of CAD. Optimal features were selected by a genetic algorithm. A multi-step balancing approach was used to detect the coronary artery. The nested ensemble had three levels, such as stacking, bagging, and vote, which classified the coronary artery based on the selected features. The simulation result showed that the model achieved high accuracy compared with other methods. However, it took more time to select features, which increased latency.

The authors in Yao et al. (2020) proposed coronary artery disease detection using machine learning algorithms. The artery features were extracted from the images using QT interval. Tunable Q wavelet transform (TQWT) and Heart rate variability (HHT) were proposed for feature decomposition. The Heart rate variability (HRV) and decomposition features were used for classification. The authors applied SVM, XGBoost, and GNB to detect coronary artery disease. The simulation result showed that these models achieved high performance in terms of accuracy, sensitivity, and specificity compared with other methods. However, these models were only suitable for small-scale environments because they required much training time in large-scale environments, which increased latency and reduced the accuracy of the process. Table 1 provides a comparison of models used for image classification and CAD diagnosis.

**Table 1:** Summary of related studies.

Ref.	Data type	Methods	Results
Wang et al. (2020a)	angiographic videos	adding a 3D convolutional layer to the input layer of the 2D CE–Net. (3D–2D CE–Net)	0.9855
Fan et al. (2018)	X-ray angiograms	multichannel FCN model (MSN-A)	0.9881
Fan et al. (2019)	X-ray angiography	hierarchical dense matching framework	Results for RMS errors of matches (pix): 9.79 ± 3.9
Jo et al. (2019)	X-ray angiography	selective feature mapping	Precision = 0.066, Recall = 0.091, specificity = 0.001, F1 score = 0.094 Acc = 0.980
Song et al. (2020)	X-ray angiographic	spatio-temporal constrained online layer separation (STOLS) method	the local and global rCNRs of the final vessel layer reached 2.54 and 1.24, respectively
Song et al. (2019)	X-ray angiographic image	novel inter/intra-frame constrained vascular segmentation method	Pre = 0.7378 Sen = 0.7960 F1 value = 0.7658
Wan et al. (2018)	X-ray angiography	multiscale-based adaptive Hessian-based enhancement method and a statistical region merging technique (Hessian matrix; Statistical region merging)	accuracy of 93%
Zhao et al. (2019)	X-ray angiography	automatic morphology estimation method by using directed graph construction for X-ray angiography images.	accuracy of 97.44%
Xiao, Li & Jiang (2020)	angiography CT images PASCAL VOC2012 dataset	three-dimensional U-net convolutional neural network	the dice coefficient of 0.8291
Sheng et al. (2019)	CT coronary angiography	Pre-processing: Adaptive vascular enhancement Feature extraction: automatic seed point detection Hessian matrix	0.863
Abdar et al. (2019a)	CAD datasets (Z-Alizadeh Sani and Cleveland)	novel nested ensemble nu-Support Vector Classification (NE-nu-SVC) model	accuracy of 94.66%
Yao et al. (2020)	electrocardiograph (ECG)	Gaussian naive Bayes Support vector machine XGBoost ResNet-18	h 96.16% accuracy

**Figure 3:**
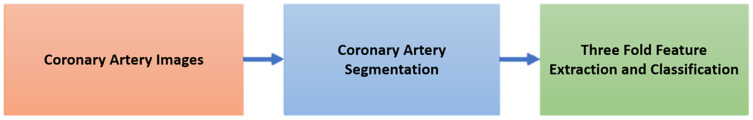
The main steps of the ASCARIS model.

The proposed ASCARIS model aimed to overcome the problems of diagnosing the coronary artery using X-ray angiograms images. Figure 3 shows the basic steps of the proposed model: (1) Owing to the attention estimator in nested U-net, the diameter of each artery can be estimated and other features useful in coronary artery disease diagnosis can be extracted; (2) a relevant feature set is extracted, consisting of the artery area, artery diameter, artery length, and artery orientation, and the angle is computed for each artery; (3) any type of artery can be segmented, *i.e*., complex, small, and overlapped, using attention-based nested U-net; (4) binarization is performed to preserve the vascular structure, which is not a higher computational method than background subtraction, and the use of lightweight algorithms improves performance with lower consumption; (5) multi-constraint-based preprocessing steps are considered for noise removal, vessel structure preservation, and contrast enhancement and binarization, resulting in morphological corrections and reducing the Mean Squared Error (MSE); (6) due to VGG16, training and testing errors can be reduced; furthermore, features can be accurately extracted in the convolutional layers, and all significant information for each coronary artery can be considered by pixel-wise processing. In this work, the authors selected VGG16 for the classification procedure, as it has several non-linear hidden layers that show complex functions effectively. We also added a dropout layer to reduce overfitting; (7) the proposed deep learning models of X-ray angiograms are suitable for any kind of patient; and (8) an optimized maximum principal curve is used to preserve the structure of vascular pixels; therefore, the accuracy of segmentation and classification is improved. In the following section, we describe the system model with the algorithms and pseudocodes used in detail.

## Materials and Methods

**Figure 4:**
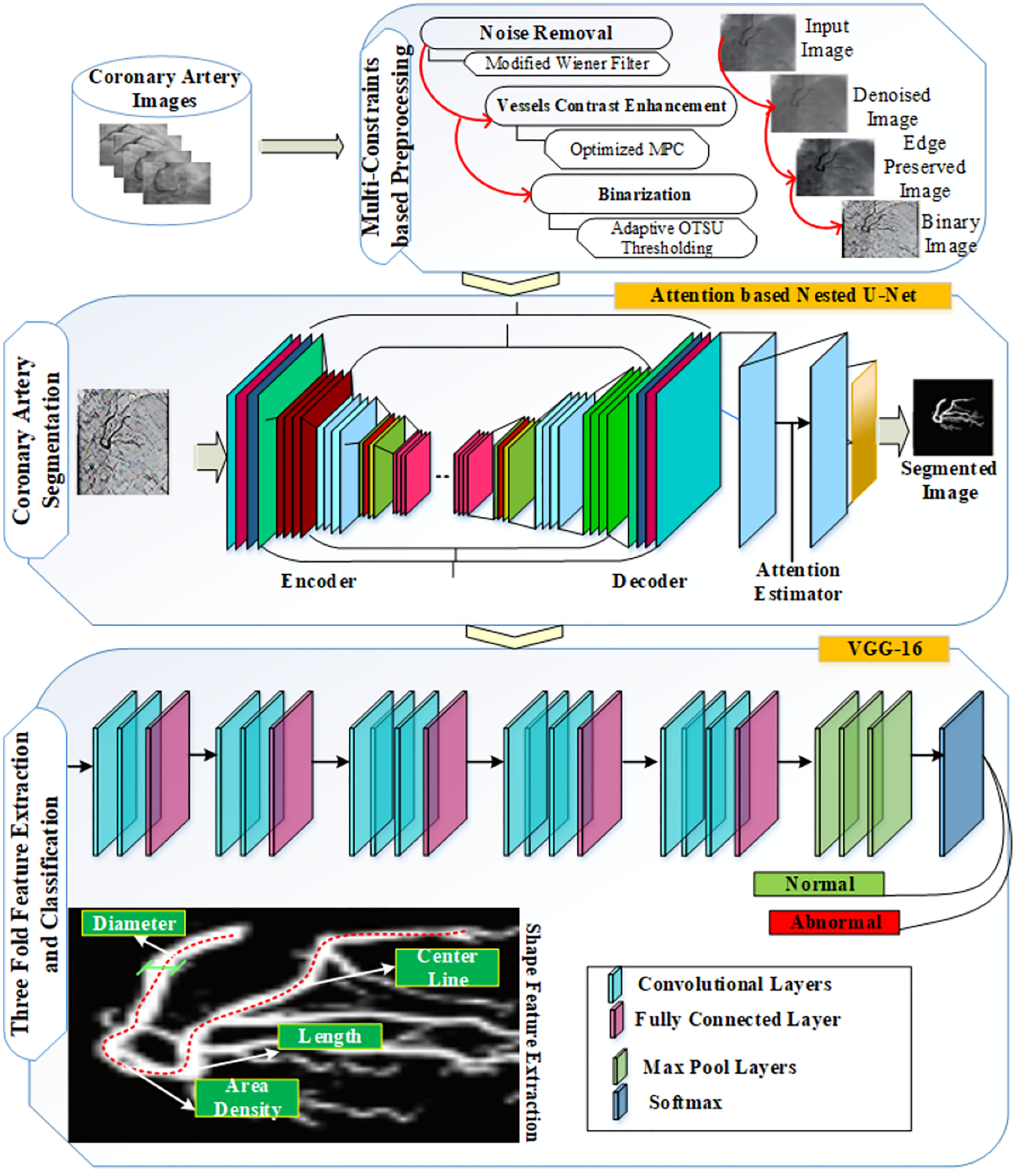
ASCARIS system model.

In this article, coronary artery disease was classified by using X-ray angiogram images. In order to predict the disease earlier, three steps were presented: multi-constraint-based preprocessing, coronary artery vessel extraction (segmentation), and threefold feature extraction and classification. Each step is illustrated in detail in the following section. The overall process of the proposed work is shown in Fig. 4.

### Multi-constant-based preprocessing

In this step, low contrast, background noise, non-uniform pixel distribution, low-quality vascular structure, and high artifacts were eliminated. In order to address these issues, multiple constraints were analyzed, and the respective operations were performed.

#### Noise removal

The first constraint was noise removal. A modified Wiener filter was used to remove both internal and external noise pixels in the image. To improve image quality, we needed to remove the noise from the input image. The mathematical representation of the noisy images was described as

(1) $$N\left( t \right) = O\left( t \right) + A\left( t \right),$$

where *N(t)* represented the noisy image in the time domain, *O(t)* represented the original images in the time domain, and *A(t)* represented the additive noise in the time domain. Detecting the noise in the input image can be very difficult, but it can easily be detected in the frequency domain; hence, converting the time domain to the frequency domain by taking the Real-Valued Fast Fourier Transform (RFFT) was required. It was defined as

(2) $$N\left( f \right) = O\left( f \right) + A\left( f \right),$$

where *O(f)* was extracted from the noisy images by multiplying the noisy images *N(f)* using the Wiener filter function *W(f)*.

(3) $$O\left( f \right) = W\left( f \right)N\left( f \right),$$

where *W(f)* represented the Wiener filter in the frequency domain, which was defined as

(4) $$W\left( F \right) = {{|O\left( F \right){|^2}} \over {|O\left( f \right){|^2} + |A\left( f \right){|^2}}},$$

where $|O\left( F \right){|^2}$ represented the original image, and $|A\left( f \right){|^2}$ represented the noisy image. The Wiener filter estimated the noisy image, which was defined as

(5) $$W\left( f \right){\approx} {{{{\left| {I\left( f \right)} \right|}^2} - {{\left| {N\left( f \right)} \right|}^2}} \over {{{\left| {I\left( f \right)} \right|}^2}}},$$

where $|I\left( f \right){|^2}$ represented the input image, and $|N\left( f \right){|^2}$ representd the noisy image. By taking the Inverse Fast Fourier Transform (IFFT), we can obtain the original image *O(t)*, which was defined as

(6) $$O\left( t \right) = IFFT\left\{ {O\left( f \right)} \right\}.$$

**Figure 5:**
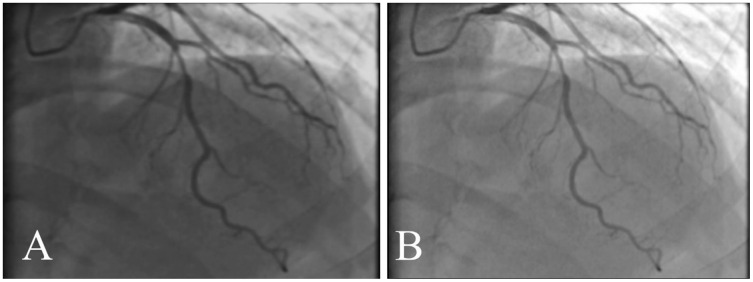
(A) X-ray image with noise. (B) X-ray image without noise.

In this manner, the noise was removed from the input image to obtain a noise-free image. The threshold for noise cancellation was adaptively set, and the filter was applied to three directions: diagonal, right, and left. Furthermore, this approach was used to recover the original image from distorted and additive uncorrelated noise (Jiang et al., 2020). Figure 5 represents the original image of coronary artery disease for noise removal.

#### Contrast enhancement

The second constraint was vessel contrast enhancement. Coronary artery vessels must be preserved before the extraction and classification processes. The loss of structural features can lead to low recognition accuracy. For structure preservation, the optimized maximum principal curvature method was used in this research to compute the global optimum pixels for the input images, which avoided sorting random pixels. The optimum set of pixels was grouped to create the structuring element in the given image. It was assumed that (x) was a noise-free image being considered for contrast enhancement. $\gamma 1,\gamma 2 \ldots .$ of the normal curvature were considered, then the minimum and maximum of the normal curvature of given a surface, known as the principal curvature. Here, the Gaussian curvature G and mean curvature $M$ were calculated based on the minimum ${\gamma ^ - }$ and maximum ${\gamma ^ + }$ principal curvature and defined as

(7) $$G = {k_{11}}{k_{22}} - k_{12}^2,$$

(8) $$M = {1 \over 2}\left( {{k_{11}} + {k_{22}}} \right),$$

where *k* represented the shape operators. The eigen values of the shape operators were defined as

(9) $$d\left( k \right) = \gamma 1\gamma 2,$$

(10) $$T\left( k \right) = \gamma 1 + \gamma 2.$$

Finally, we obtained the principle curvature values, which were defined as

(11) $${\gamma ^ - } = Min(\gamma 1,\gamma 2\} = M - \sqrt {{M^2} - G} ,$$

(12) $${\gamma ^ + } = Max(\gamma 1,\gamma 2\} = M + \sqrt {{M^2} - G} .$$

**Figure 6:**
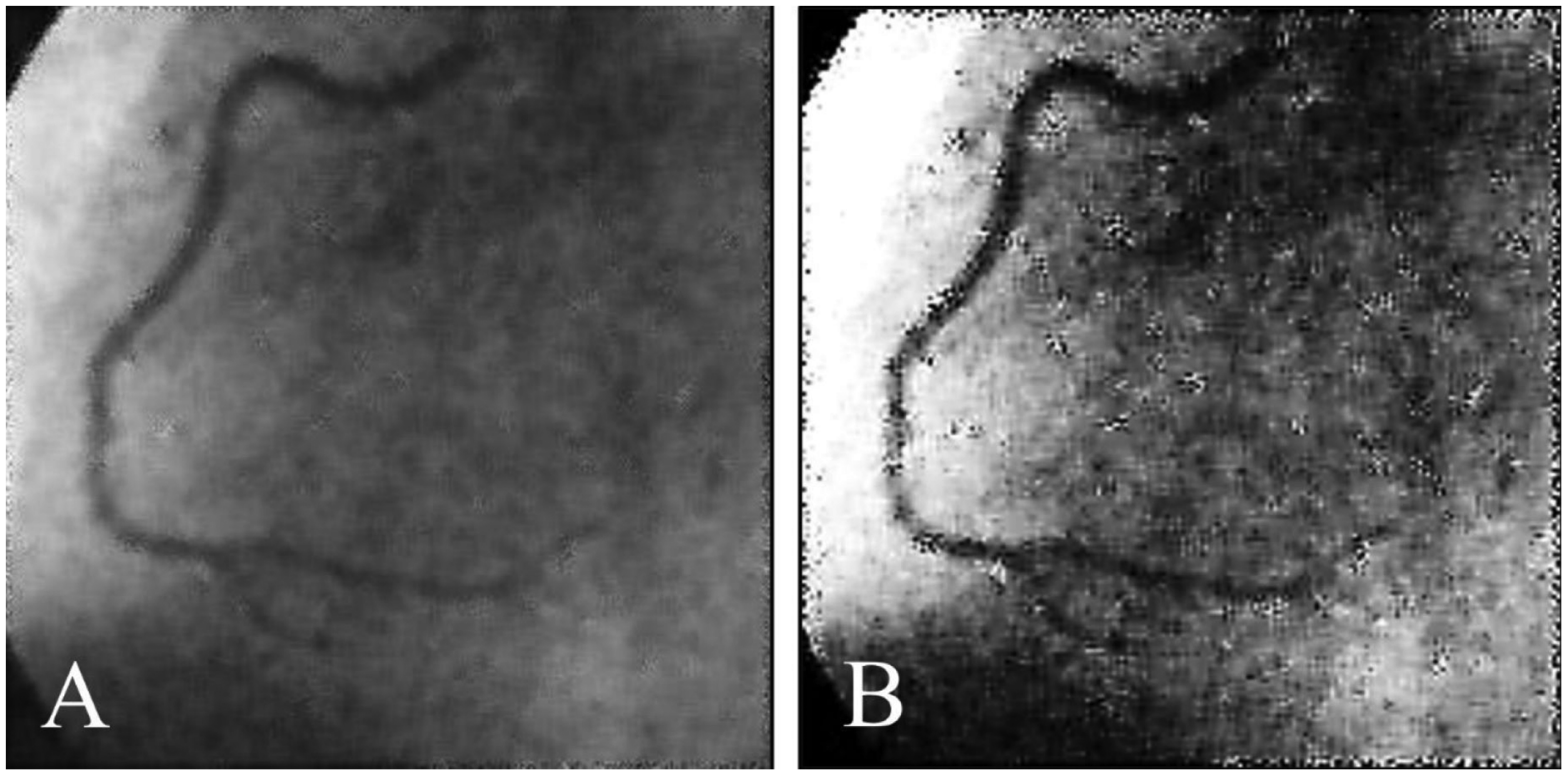
(A) Image before contrast enhancement; (B) image after contrast enhancement.

The optimized maximum principal curvature detected the edges in the background, which increases the contrast of the arteries in the image. Figure 6 shows the image before contrast enhancement and the image after contrast enhancement.

#### Binarization

The third constraint was binarization. Adaptive Otsu thresholding was used to transform the contrast-enhanced image into the binarized image. It was used to distinguish the foreground from the background. The thresholding method was used to convert the image into binary form, which reduced the complexity of the process of classification. This method worked on the image histogram. It consisted of two classes of pixels, foreground and background, which set the threshold on the image to minimize the intra-class variance. The threshold was calculated by adding the foreground and background values, and the threshold minimized the weight values within the class variance. The weighted sum of the two classes was defined as

(13) $$\alpha \left( t \right) = w1\left( t \right)\alpha 1\left( t \right) + w2\left( t \right)\alpha 1\left( t \right),$$

where $w1$ and $w2$ represented the weight values of the two classes, and $\alpha 1\left( t \right)$, $\alpha 2\left( t \right)$ represented the threshold variances of the two classes. The probability of class was calculated from the histograms and defined as

(14) $$w1\left( t \right) = \sum\limits_{i = 0}^{t - 1} s \left( i \right),$$

(15) $$w2\left( t \right) = \sum\limits_{i = t}^{l - 1} s \left( i \right).$$

The value of the minimized intra-class variance was equal to the maximum inter-class variance and defined as

(16) $$\alpha k\left( t \right) = {\alpha ^2} - {\alpha ^2}w\left( t \right) = w1{\left( {\mu 1 - \mu n} \right)^2} + w2{\left( {\mu 2 - \mu n} \right)^2}$$

(17) $$= w1\left( t \right)w1\left( t \right){\left[ {\mu 1\left( t \right) - \mu 2\left( t \right)} \right]^2},$$

where $\mu$ represented the class means, and $\mu 1\left( t \right),\mu 2\left( t \right),\;{\rm{and}}\;\mu n(t)$ were listed as

(18) $$\mu 1\left( t \right) = {{\sum\limits_{i = 0}^{t - 1} i s\left( i \right)} \over {w1\left( t \right)}},$$

(19) $$\mu 2\left( t \right) = {{\sum\limits_{i = 0}^{L - 1} i s\left( i \right)} \over {w2\left( t \right)}},$$

(20) $$\mu n\left( t \right) = \sum\limits_{i = 0}^{t - 1} i s\left( i \right),$$

where

(21) $$w1\mu 1 + w2\mu 2 = \mu n,$$

(22) $$w1 + w2 = 1.$$

**Figure 7:**
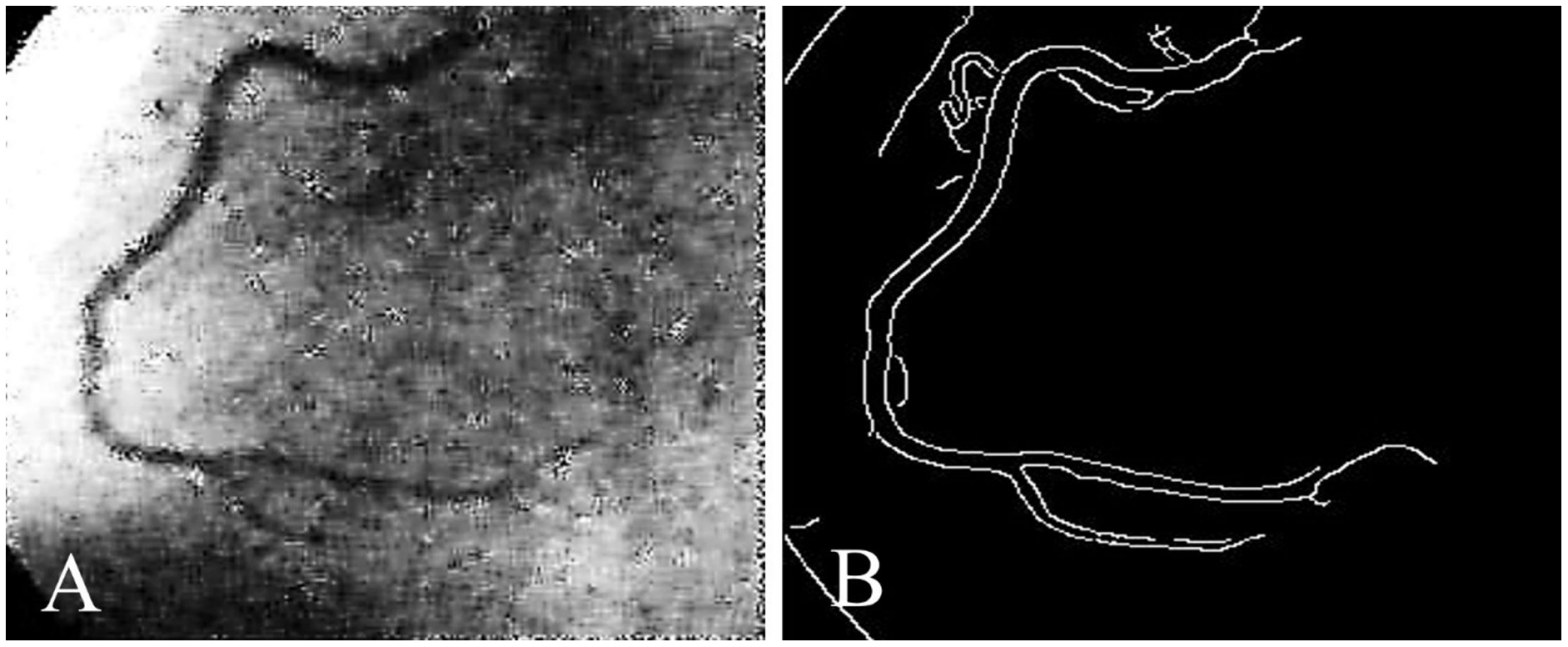
(A) Contrast enhanced image; (B) binarized image.

The threshold values were calculated for the binarized image, which reduced the complexity of the process and increased image quality. The threshold value was adaptively changed to the corresponding images. Figure 7 represents the binarized and contrast enhancement images.

### Coronary artery vessel extraction

In this step, the coronary artery was extracted using an attention-based nested U-net. This is the modified version of nested U-net. By considering each artery, it segments all the arteries in the image. With this step, cardiologists can quantify and classify stenosis, a type of heart disease. The diameter of the coronary artery is about 5 mm, and each slice’s image resolution can be 512 $\times$ 512 voxels. The image view is 20 cm $\times$ 20 cm. Small coronary arteries occupy 50 voxels on the slice, and the large coronary artery is 500 voxels. However, coronary artery extraction is sometimes difficult when the vessels intersect, overlap, and cross in the X-ray angiogram images. False positive rate reduction is a challenging task for small objects. To improve classification accuracy, current segmentation frameworks do not rely on a variability in segmentation. Furthermore, bones overlap the coronary artery. The artery angle estimation-based segmentation method was presented in this research, which ran on a nested U-net. In this article, we achieved this goal by including an attention gate in the standard nested U-net. The attention gate learned and extracted the significant characteristics of the binarized image. In this method, we considered three important features of the coronary artery for segmentation. Table 2 lists the features extracted from the images:

- Diameter
- Density
- Tube-like features (shape, size, *etc*.)

**Table 2:** Extracted features.

Color features	Mean Standard deviation Skewness Local intensity Kurtosis
Texture features	Entropy Correlation Homogenity ASM
Shape features	Artery diameter Artery area Artery angle Artery length Eccentricity Roundness Dispersion Convexity Solidity

**Figure 8:**
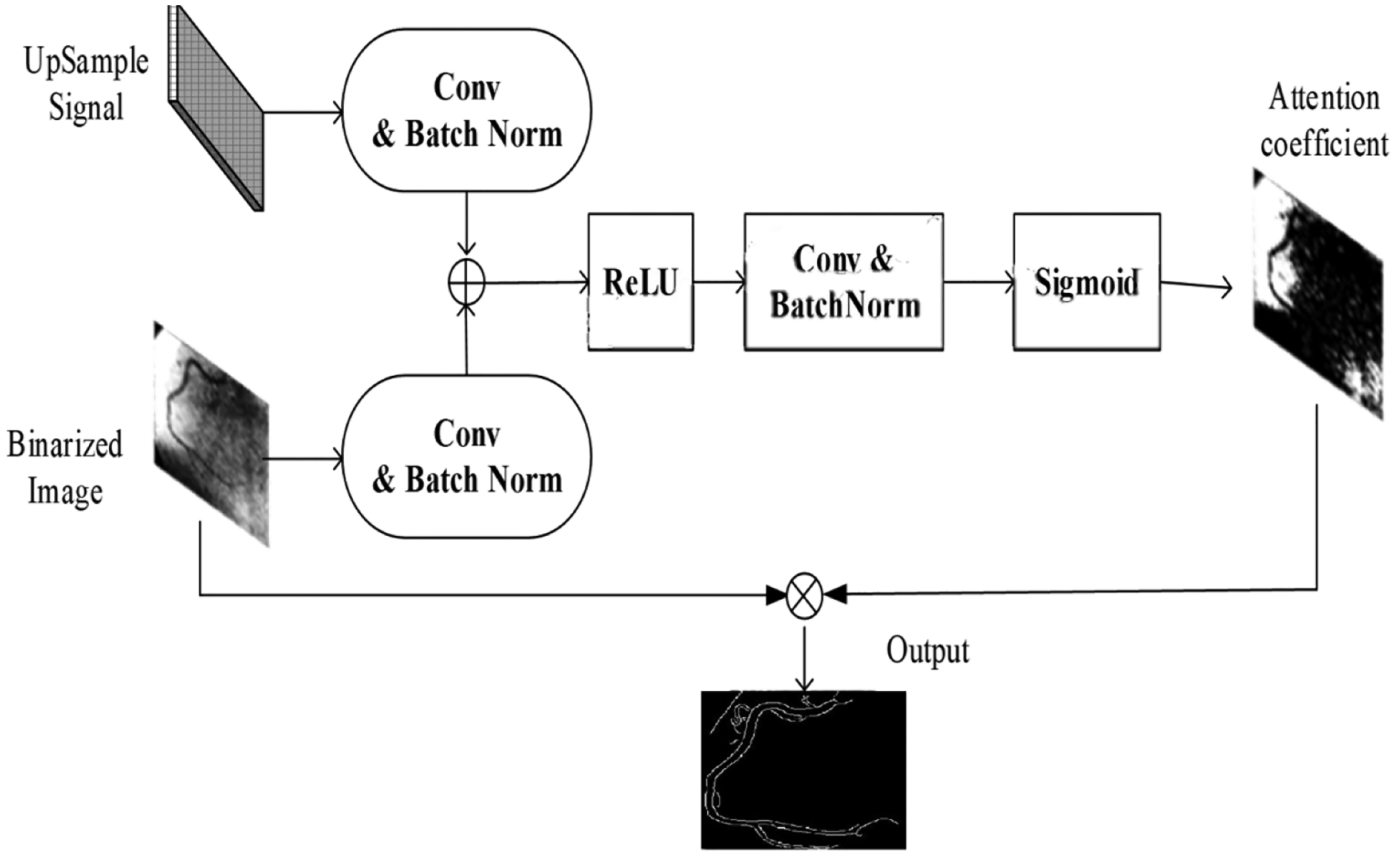
Attention gate architecture.

However, determining these characteristics of the coronary artery is a challenging task, since density is identified by the contrast agent dose and the precision of the equipment. Here, the attention gate was used to estimate the important characteristics of the coronary artery. The input for coronary artery extraction was the binarized image. In the attention-based U-net model, bitwise and operation were applied to the binary images and original image. The resulting image was a segmented portion of the image. Figure 8 depicts the attention gate for the nested U-net.

Compared with the nested U-net, the attention gate provides several benefits:

- At any depth and density, the shapes and sizes of the arteries can be extracted by learning, thus reducing the complexity in the extraction of the arteries’ statistical features.
- To learn the importance of the coronary arteries characteristics, the nest with attention gate uses a feature extractor that performs the training of the nested U-net.
- To obtain different levels of features for the coronary arteries, various decoder paths can be used by individuals, and end masks can be generated for different coronary arteries.

In order to focus on the coronary arteries (target entities), the attention gate uses the upsampling feature $G$ decoder and the corresponding depth feature $F$ in the encoder. At first, the gating signal enhanced the second input learning $F$ and forwarded it to the upward decoder. After convolutional operation and batchnorm, the gating signal and encoded features were combined pixel by pixel. Then, the merged result was forwarded to the rectified linear unit (ReLU, ${\sigma _1}\left( x \right) = \max (0,x)$, for activation. Convolutional operation and batchnorm were applied to the binarized image, which were represented by ${W_\theta }$ and ${b_\theta }$, respectively. It was assumed that the S-shaped activation function may be applied to these two norms, and the sigmoid operation can be defined as follows:

(23) $${\sigma _2}\left( x \right) = {1 \over {1 + {e^{ - x}}}}.$$

The final output can be processed by the attention gate coefficient $\alpha$, and the process of the attention gate for coronary artery segmentation can be defined as follows:

(24) $$f = {\sigma _1}\left[ {W_F^t \times F + {b_F}} \right] + \left[ {W_G^t \times G \times {b_G}} \right],$$

(25) $$\alpha = {\sigma _2}\left( {W_\theta ^T \times F + {b_\theta }} \right),$$

(26) $$Output = F*\alpha.$$

The attention gate was a good module that ran between the U-net layers, and the proposed model improved the propagation semantic features *via* skip connection functionality.

### Threefold feature extraction and classification

After segmentation, the features were extracted from each artery. The main categories of features were color, shape, and texture. The feature selection phase eliminated irrelevant features while retaining basic discriminant information, thus helping the data extraction process effectively and improving the quality of the data set and the performance of the deep learning systems. In this process, the VGG16 architecture was used for both feature extraction and classification, which falls under deep learning algorithms. First, it extracted the features from the segmented region. The architecture of VGG16 consisted of multiple convolution layers with max pooling layers between the convolution layers, followed by three fully connected (dense) layers. The output layer with SoftMax was the activation function. VGG16 can extract features at a low level using a small kernel size, and it also has lower layer numbers compared with VGG19. The max pooling and average pooling layers were used to extract the features from the image. The layers extracted from the segmented region provided color features (mean, standard deviation, skewness, local intensity, and kurtosis), texture features (such as entropy, correlation, homogeneity, ASM, and entropy), and shape features (namely artery diameter, artery area, artery angle, and artery length, and also eccentricity, roundness, dispersion, convexity, and solidity).

The diameter of the artery was an important feature for classifying the CAD images. The artery diameter can be formulated as

(27) $${D_m} = \sqrt {{{\left( {{x_1} - {x_2}} \right)}^2} + {{\left( {{y_1} - {y_2}} \right)}^2}} ,$$

where the coordinates P ( ${x_1},{y_1}$) and Q ( ${y_1},{y_2})$ were points on opposite sides of the artery. Then, the result of the max pool and average pooling were concatenated and sent to the convolution layer with a size of $7 \times 7$ by sigmoid function $(\delta )$. The result of the convolutional layer was defined as

(28) $$C\left( F \right) = \delta \left( {{f^{7 \times 7}}\left[ {{F_A};{F_M}} \right]} \right),$$

where ${F_A} \in {r^{1 \times h \times w}}$ and ${F_M} \in {r^{1 \times h \times w}}$ represented the results obtained by average pooling and max pooling, respectively, while h represented the height and w the width of the layer.

**Figure 9:**
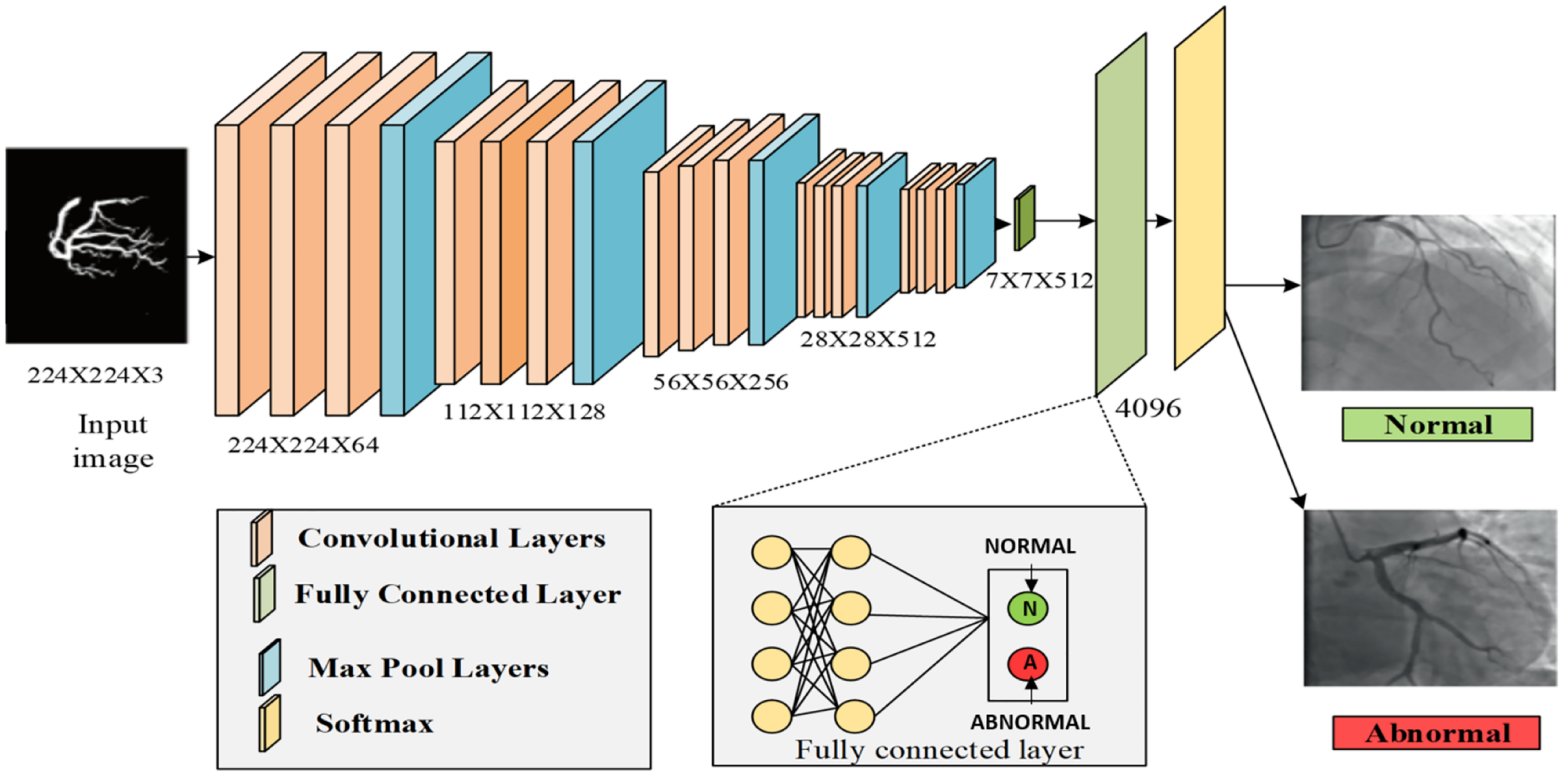
Process of VGG-16.

Then, the convolutional layers had the extracted features, which were known as the fourth pooling layer of the proposed VGG16 model. The extracted results were forwarded to the fully connected layer, which consisted of three layers: the dropout, flatten, and dense layers. To classify the features, VGG16 used the SoftMax layer, which was the last dense layer. It classified the features by using the attention estimator to compute the weight value based on the feature’s importance. The input image was classified as normal or abnormal (affected by CAD). The classification result was defined as

(29) $$C\left( {p = {q \over s}} \right) = {{{e^b}} \over {\sum\limits_j {{e^{{b_j}}}} }},$$

where *q* and *s* represented the probabilities that were collected from the SoftMax layer. Finally, the results were classified into normal and abnormal classes. Figure 9 represents the process of VGG16. Table 3 explains the proposed architecture for VGG16. Table 4 explains the pseudocode of VGG16.

**Table 3:** Architecture for VGG-16.

Input size	Layer	Kernel	Stride	Output size
224 $\times$ 224 $\times$ 3	Conv1-64	3 × 3	1	224 $\times$ 224 $\times$ 64
224 $\times$ 224 $\times$ 64	Conv1-64	3 × 3	1	224 $\times$ 224 $\times$ 64
224 $\times$ 224 $\times$ 64	maxpool	2 × 2	2	112 $\times$ 112 $\times$ 64
112 $\times$ 112 $\times$ 64	Conv2-128	3 × 3	1	112 $\times$ 112 $\times$ 128
112 $\times$ 112 $\times$ 128	Conv2-128	3 × 3	1	112 $\times$ 112 $\times$ 128
112 $\times$ 112 $\times$ 128	maxpool	2 × 2	2	56 $\times$ 56 $\times$ 128
56 $\times$ 56 $\times$ 128	Conv3-256	3 × 3	1	56 $\times$ 56 $\times$ 256
56 $\times$ 56 $\times$ 256	Conv3-256	3 × 3	1	56 $\times$ 56 $\times$ 256
56 $\times$ 56 $\times$ 256	Conv3-256	3 × 3	1	56 $\times$ 56 $\times$ 256
56 $\times$ 56 $\times$ 256	maxpool	2 × 2	2	56 $\times$ 56 $\times$ 256
56 $\times$ 56 $\times$ 256	Conv4-512	3 × 3	1	28 × 28 × 256
28 × 28 × 256	Conv4-512	3 × 3	1	28 × 28 × 512
28 × 28 × 512	Conv4-512	3 × 3	1	28 × 28 × 512
28 × 28 × 512	maxpool	2 × 2	2	28 × 28 × 512
28 × 28 × 512	Conv5-512	3 × 3	1	14 × 14 × 512
$14 \times 14 \times$ 512	Conv5-512	3 × 3	1	14 × 14 × 512
$14 \times 14 \times$ 512	Conv5-512	3 × 3	1	14 × 14 × 512
$14 \times 14 \times$ 512	maxpool	2 × 2	2	14 × 14 × 512
1 $\times\; 1\; \times$ 25,088	fc	1 × 1	–	1 $\times\; 1 \times 4,096$
1 $\times\; 1 \times 4,096$	fc	1 × 1	–	1 $\times\; 1 \times 4,096$
1 $\times\; 1 \times 4,096$	fc	1 × 1	–	1 $\times\; 1 \times 1,000$

**Table 4:** Pseudocode of VGG-16.

Pseudocode: VGG-16
1. Input: Segmented region (SR)
2. Output: Normal (N) or abnormal (AN)
3. {
4. Begin
5. Initialize $\{ fc,fs,ft\}$
6. $fc \leftarrow \{ fc1,fc2,..fcn\}$ // color features
7. $fs \leftarrow \{ fs1,fs2,..fsn\}$ //shape features
8. $ft \leftarrow \{ ft1,ft2,..ftn\}$ //texture features
9. Initialize VGG-16
10. VGG-16 $\leftarrow$ raw training data send to VGG-16 for featured extraction
11. for $i \leftarrow o\ to\ n$ do
12. Extract $fc$ from SR
13. Extract $fs$ from SR
14. Extract $ft$ from SR
15. $F \leftarrow \{ fc,fs,ft\}$
16. Extract the features by average pooling layer ${F_A}$
17. Extract the features by maxpool layer ${F_M}$
18. Concatenate the features using Eq. (28)
19. Classifying the images using Eq. (29) by softmax layer
20. Class $\leftarrow \{ N,AN\}$
21. End for
22. }
23. Return class
24. End
25. }

## Results

### Experimental study

In this section, experimentation of the proposed ASCARIS model was carried out in order to evaluate the performance of the approach. This section is divided into three sub-sections, namely simulation setup, comparative analysis, and discussion.

#### Environmental setup

The simulation of the proposed ASCARIS model was executed in the MATLAB R2020a simulation tool, which supported processes such as the preprocessing, segmentation, and classification of segmented images for the detection of CAD. The system requirements for the simulation of the proposed model are presented in Table 5.

**Table 5:** System requirements.

Software requirements	Matlab	R2020a
	OS	Windows 10 pro
Hardware requirements	RAM	8 GB
	CPU	2.90 GHZ
	Hard disk	1 TB
	Processor	Intel core

The segmentation of coronary arteries for the purpose of classification was performed by means of the attention-based nested U-net, whereas the existing approaches utilize the conventional U-net, which decreases classification accuracy. The extraction of threefold features and the classification of images were carried out by utilizing VGG16.

**Dataset Description**: The set of coronary artery images (X-ray coronary angiography images) were collected from a clinical database. The blood vessels in the images were of uneven thickness and complicated in vascular structure by the background. Furthermore, the coronary artery image set contained many noises. The dataset used in this research contained 130 X-ray coronary angiograms. Their corresponding ground truth images were available in portable gray map (PGM) format, and each coronary artery image size was 300 × 300 pixels. It was gathered from the cardiology department of the Mexican Social Security Institute. UMAE T1-Leon provided this medical database for heart disease diagnosis. Ethical approval for the use of the database was obtained for this article under reference number R-2019-1001-078.

In this article, the data set was divided into two parts at random. The first section used to train the model contained 100 images. The second section contained 30 images to test the proposed model based on the features of color, diameter, and shape.

The database source used in this research can be accessed through the official link, http://personal.cimat.mx:8181/~ivan.cruz/DB_Angiograms.html.

#### Comparative analysis

The validation of the proposed ASCARIS model was carried out in an extensive manner in which a comparison of the proposed model with existing approaches was performed in terms of performance metrics, such as accuracy, sensitivity, specificity, revised contrast-to-noise ratio, mean square error, dice coefficient, Jaccard similarity, Hausdorff distance, PSNR, segmentation accuracy, and ROC curve.

##### Accuracy

Accuracy is a significant metric in evaluating the performance of an approach. The accuracy of this approach can be computed as

(30) $$Acc = {{T{r_p} + T{r_n}} \over {NP + NN}},$$

where $T{r_p}\;{\rm{and}}\;{{T}}{{{r}}_{{n}}}$ denote the number of positive and negative images detected accurately, and $NP\;{\rm{and}}\;{{NN}}$ denote the total number of positive and negative images, respectively.

**Figure 10:**
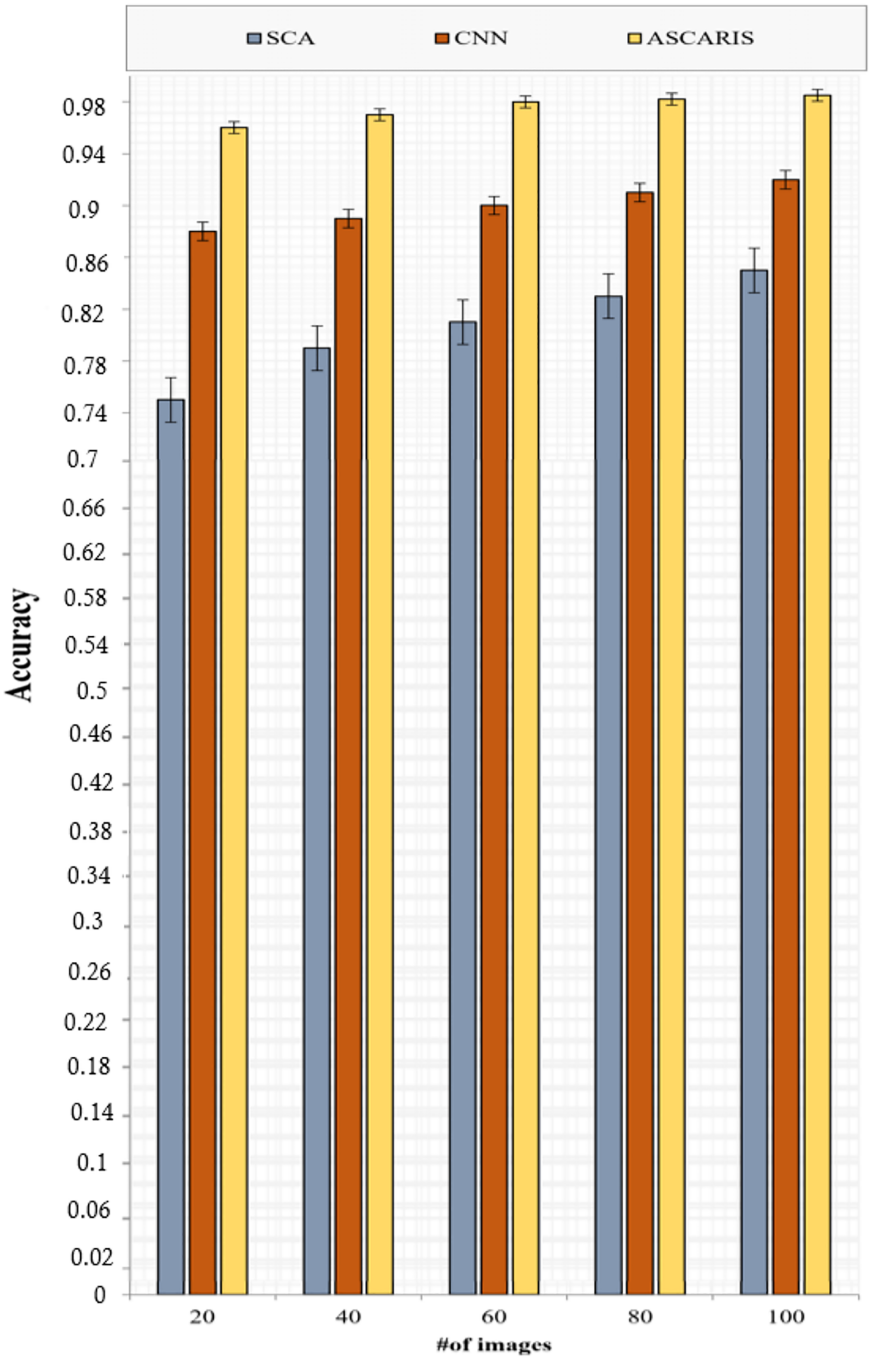
Accuracy *vs* # of images.

Figure 10 depicts the analysis of the accuracy of the proposed ASCARIS model and the existing approaches with respect to the number of images. The accuracy of the proposed model was higher than the existing approaches due to the extraction of more important features using the attention layer in the U-net, which contributed to the increased accuracy of the classification process. The model achieved 97% accuracy. The existing approaches performed classification based on fixed diameters, which affected the accuracy. A lack of consideration of essential parameters for the segmentation of the arteries resulted in a degradation of accuracy.

##### Sensitivity

**Figure 11:**
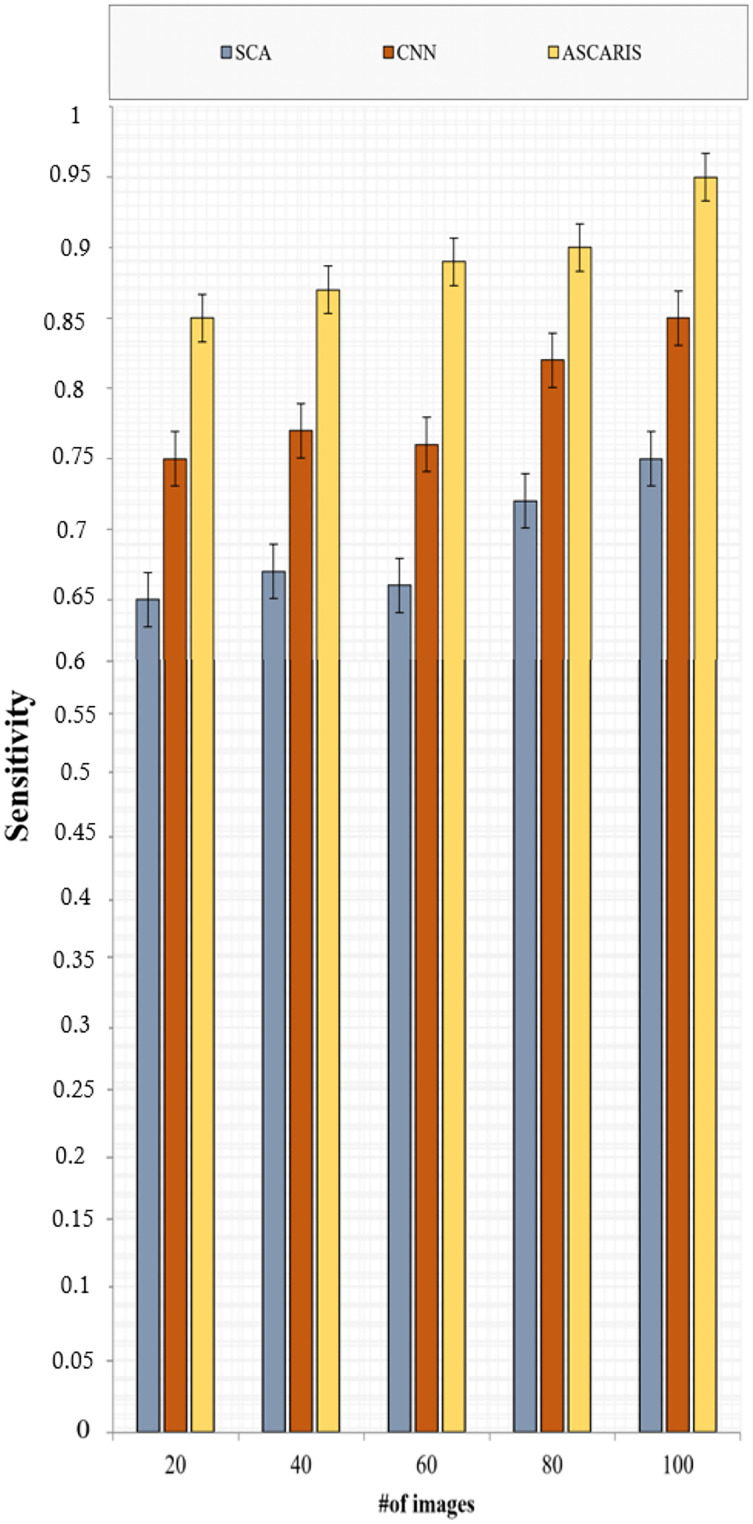
Sensitivity *vs* # of images.

The term sensitivity is also called recall. It is defined as the measure of relevancy from the detected result. The sensitivity can be formulated as

(31) $$SN = {{T{r_p}} \over {T{r_p} + F{l_n}}},$$

where $F{l_n}$ denotes the number of non-diseased X-rays that were classified as diseased. Figure 11 illustrates the comparison of sensitivity of the proposed ASCARIS model with the existing models with respect to the number of images. The sensitivity of the proposed approach was high due to the classification of X-ray images based on the threefold feature extraction in which three categories of features, such as color, texture, and shape, were analyzed to determine coronary artery disease. The model achieved 95% sensitivity. The existing approaches classified the testing images only based on shape features. They were not as effective in identifying CAD in small arteries, thereby reducing sensitivity.

##### Specificity

**Figure 12:**
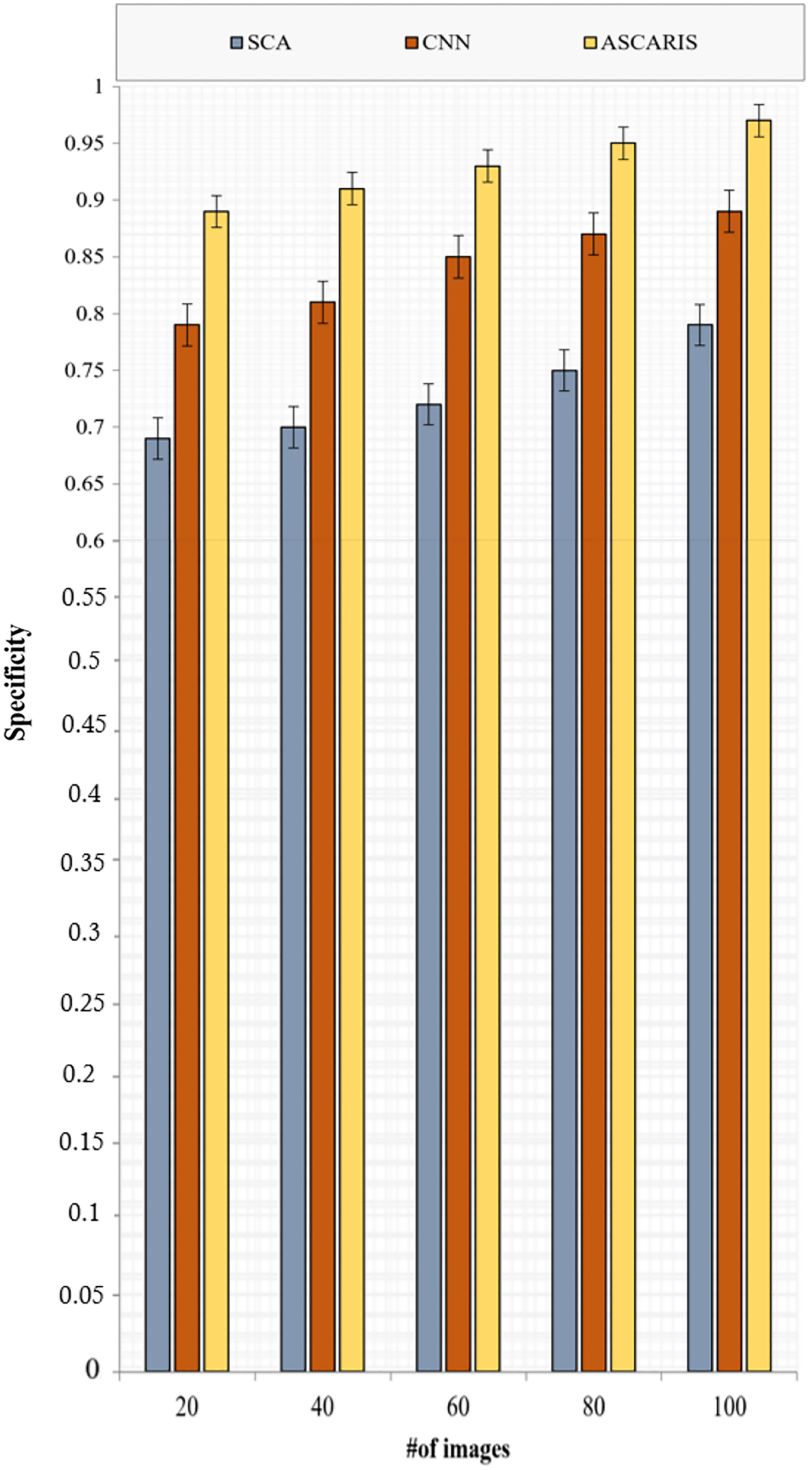
Specificity *vs* # of images.

Specificity is defined as the measure of correctness in the detected set of outputs. The specificity of this approach can be formulated as

(32) $$SN = {{T{r_n}} \over {T{r_n} + F{l_p}}},$$

where $F{l_p}$ denotes the false positive class in which the diseased images were classified as normal. Figure 12 depicts the specificity analysis of the proposed ASCARIS model and the existing approaches with respect to the number of images. The proposed approach was found to possess high specificity due to the estimation of the angle of arteries for the purpose of segmentation, which precisely segmented the arteries even in overlapped conditions. The model achieved 93% specificity. This further contributed to the increased specificity of the classification process. The existing approaches performed inefficient techniques for the purpose of segmentation, which made it difficult to differentiate the arteries for the purpose of detecting diseased arteries.

##### Revised contrast-to-noise ratio

**Figure 13:**
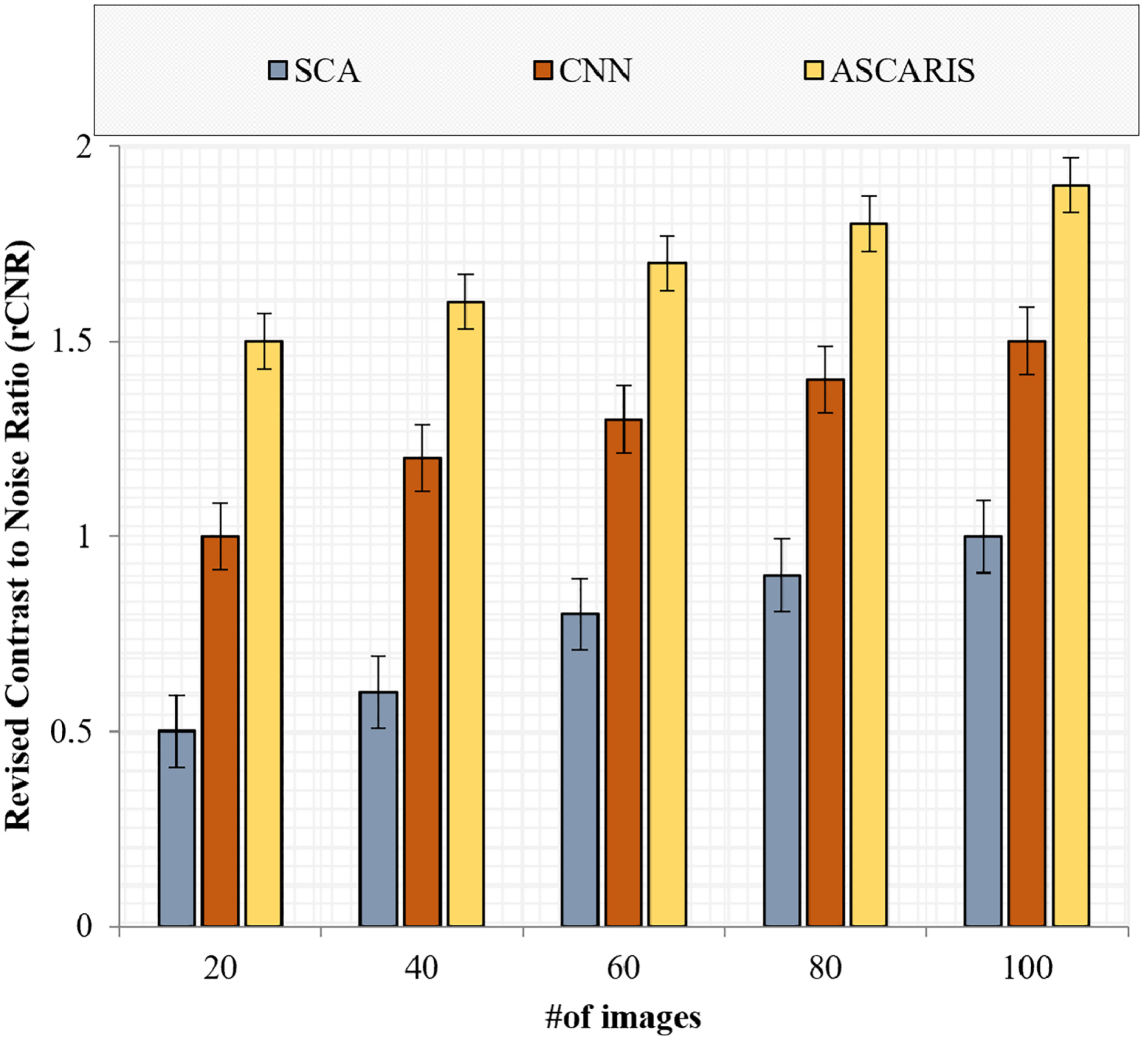
Revised contrast to noise ratio *vs* # of images.

The revised contrast-to-noise ratio (rCNR) is an important metric to validate the images by assessing the quality of the images. The rCNR refers to the amount of contrast present between the background and the target artery. The quality of the image is good only when the rCNR is high. In some cases, the Signal-to-noise ratio (SNR) of an image will be high, but the features of the images may not be efficient due to insufficient contrast; therefore, the rCNR is computed for images. The determination of the rCNR of an image can be formulated as

(33) $$rCNR = {{\left| {I{n_A} - I{n_B}} \right|} \over {{\sigma _n}}},$$

where $I{n_A}\;{\rm{and}}\;{{I}}{{{n}}_{{B}}}$ denote the intensities of the arteries and the background in the image, respectively, and ${\sigma _n}$ denotes the quantity of noise in the image. Figure 13 depicts the comparison of the rCNR of our proposed ASCARIS approach and the existing approaches with respect to the number of images. The proposed approach possessed a higher rCNR value (1.7 $\pm \; 0.02$) due to the implementation of preprocessing in which the enhancement of contrast was performed by utilizing the optimized maximum principal curvature method, thereby improving the contrast by preserving the edge details. The existing approaches lacked the proper contrast enhancement technique, thereby possessing a low rCNR value.

##### Mean square error

**Figure 14:**
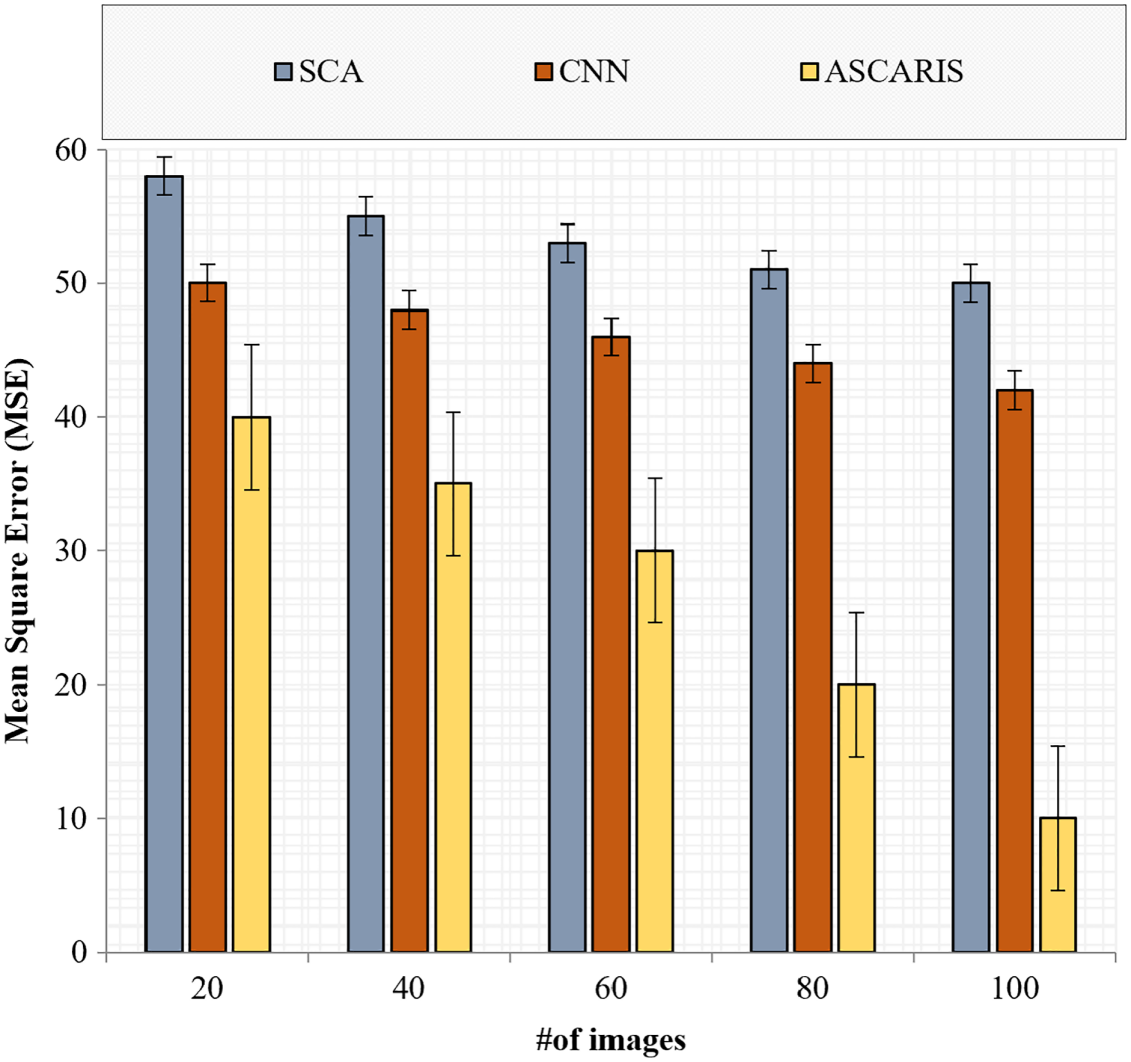
Mean square error *vs* # of images.

The mean square error (MSE) is a significant metric in analyzing the efficiency of a classification. It is defined as the measure of closeness in classification. The lower the value of the MSE, the higher the efficiency of the classification approach. The MSE of an approach can be computed as

(34) $$MSE = {1 \over N}\sum\limits_{k = 1}^N {{{\left( {{R_i} - {{\hat R}_i}} \right)}^2}} ,$$

where $N$ denotes the total number of images, and ${R_i}{\;{\rm and}}\;{\hat R_i}$ denote the actual result and classified result, respectively. Figure 14 illustrates the comparison of the MSE of the proposed ASCARIS model with the existing models with respect to the number of images. The MSE of the proposed approach was lower than the existing approaches (27.2 $\pm \; 0.1$) due to the increased accuracy of our approach. VGG16 performed the classification better with deep CNN architecture, thereby reducing the MSE. The existing approaches performed the classification using a deep CNN model, which was not efficient in extracting the relationship between the features and resulted in a higher MSE.

##### Dice coefficient

**Figure 15:**
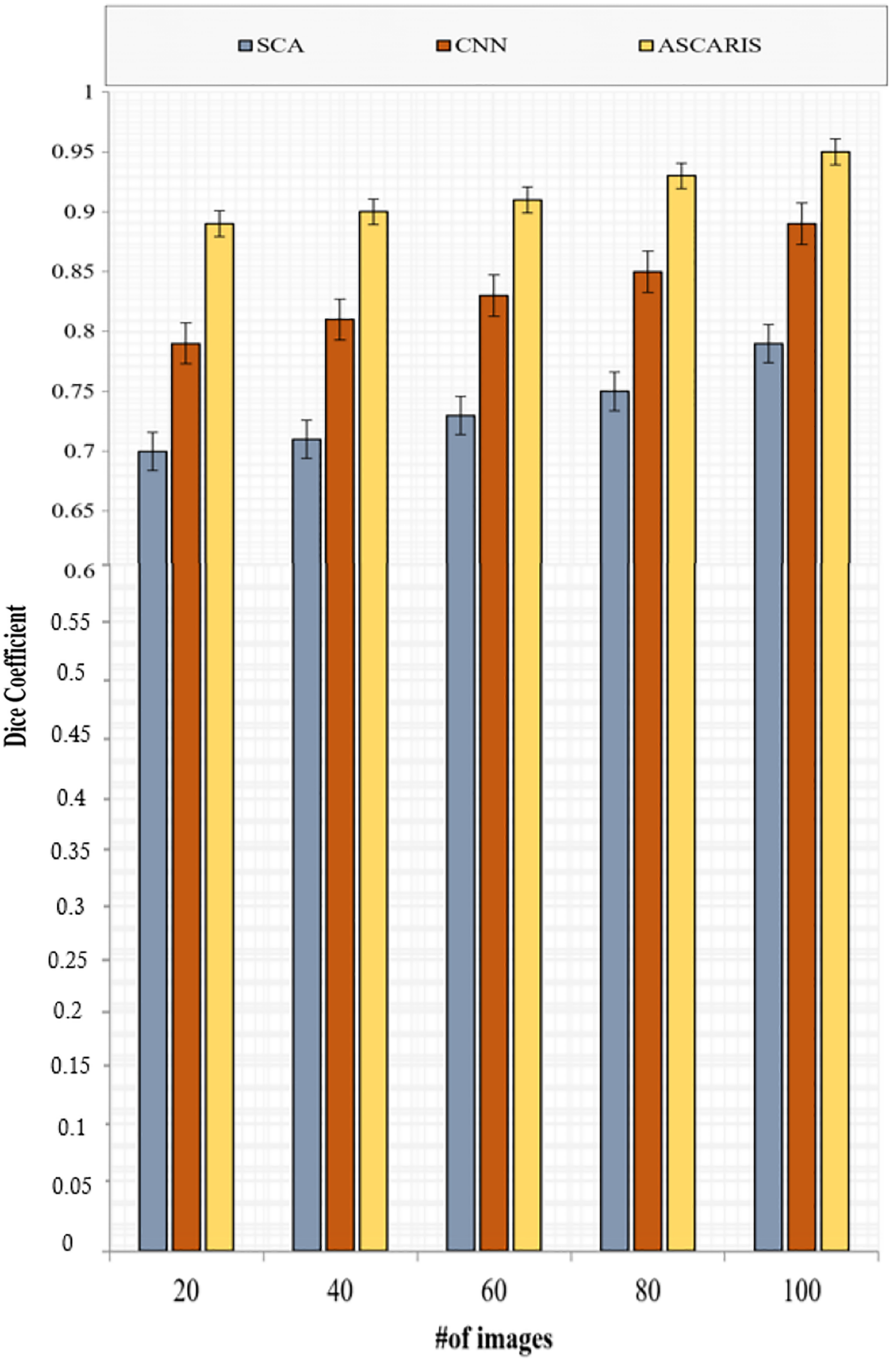
Dice coefficient *vs* # of images.

The dice coefficient is similar to precision, which is used to determine the spatial overlap of segmented images. The value of the dice coefficient ranges from 0 to 1. The dice coefficient can be formulated as

(35) $$DC = {{2\left| {{R_ + } \cap {{\hat R}_ + }} \right|} \over {\left| {{R_ + }} \right| + \left| {{{\hat R}_ + }} \right|}},$$

where ${R_ + }\;{\rm{and}}\;{\hat R_ + }$ denote the actual and segmented parts of the arteries. Figure 15 depicts the comparison of the dice coefficient of the proposed ASCARIS model with the existing approaches with respect to the number of images. The DC of our proposed approach was higher than the existing approaches due to the execution of the angle estimation of arteries and the implementation of the attention estimator for selecting the significant features in order to perform the segmentation. The existing approaches possessed low dice coefficients due to the lack of consideration of the artery angle.

##### Jaccard similarity

Jaccard similarity is computed in order to measure the similarity between a detected output and an original diseased image. Jaccard similarity can be computed as

(36) $$JS = {{\left| {{R_ + } \cap {{\hat R}_ + }} \right|} \over {\left| {{R_ + }} \right| \cup \left| {{{\hat R}_ + }} \right|}}$$

**Figure 16:**
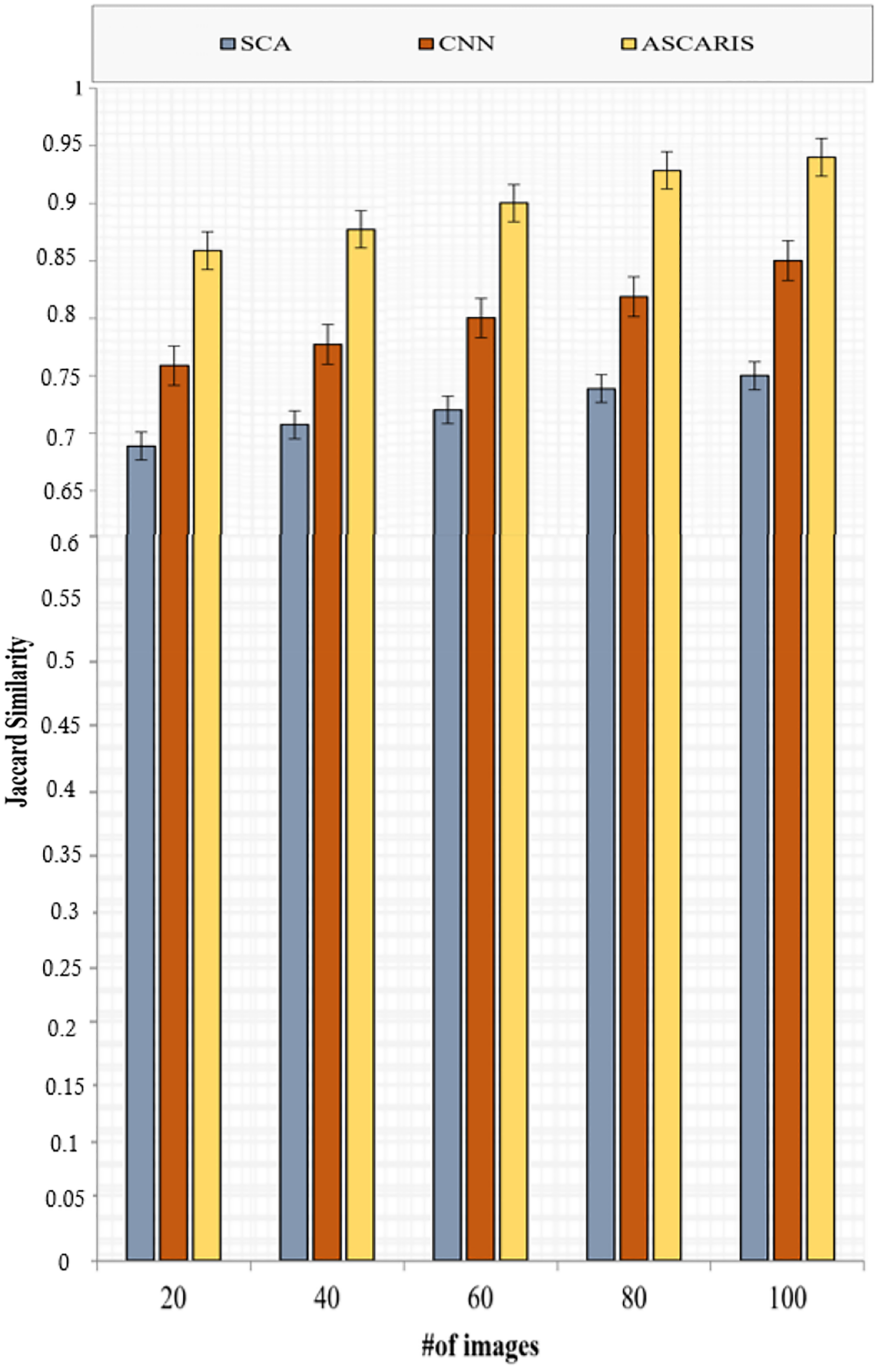
Jaccard similarity *vs* # of images.

Figure 16 depicts the analysis of the Jaccard similarity of the proposed approach and the existing approaches with respect to the number of images. The Jaccard similarity of our proposed approach was high (89%) due to the classification based on the threefold extraction of features. These features provided all the significant measures to identify the diseased images. The classification of images using the VGG16 architecture improved the accuracy of the classification, thus improving Jaccard similarity. The existing approaches lacked the extraction of significant features required for classification, which reduced the value of Jaccard similarity.

##### Hausdorff distance

The Hausdorff distance is an important metric to validate the segmentation of images. It is the measure of correctness in segmenting an image. The Hausdorff distance can be formulated as

(37) $$Di{s_H}\left( {P,Q} \right) = max\left| {{d_{PQ}},{d_{QP}}} \right| = max\left\{ {{{\max_{p \in P}}}\;{{\min_{q \in Q}}\; d\left( {p,q} \right),\;{{\max_{q \in Q}}}\;{{\min_{p \in P}}}}\; d\left( {p,q} \right),} \right\}.$$

**Figure 17:**
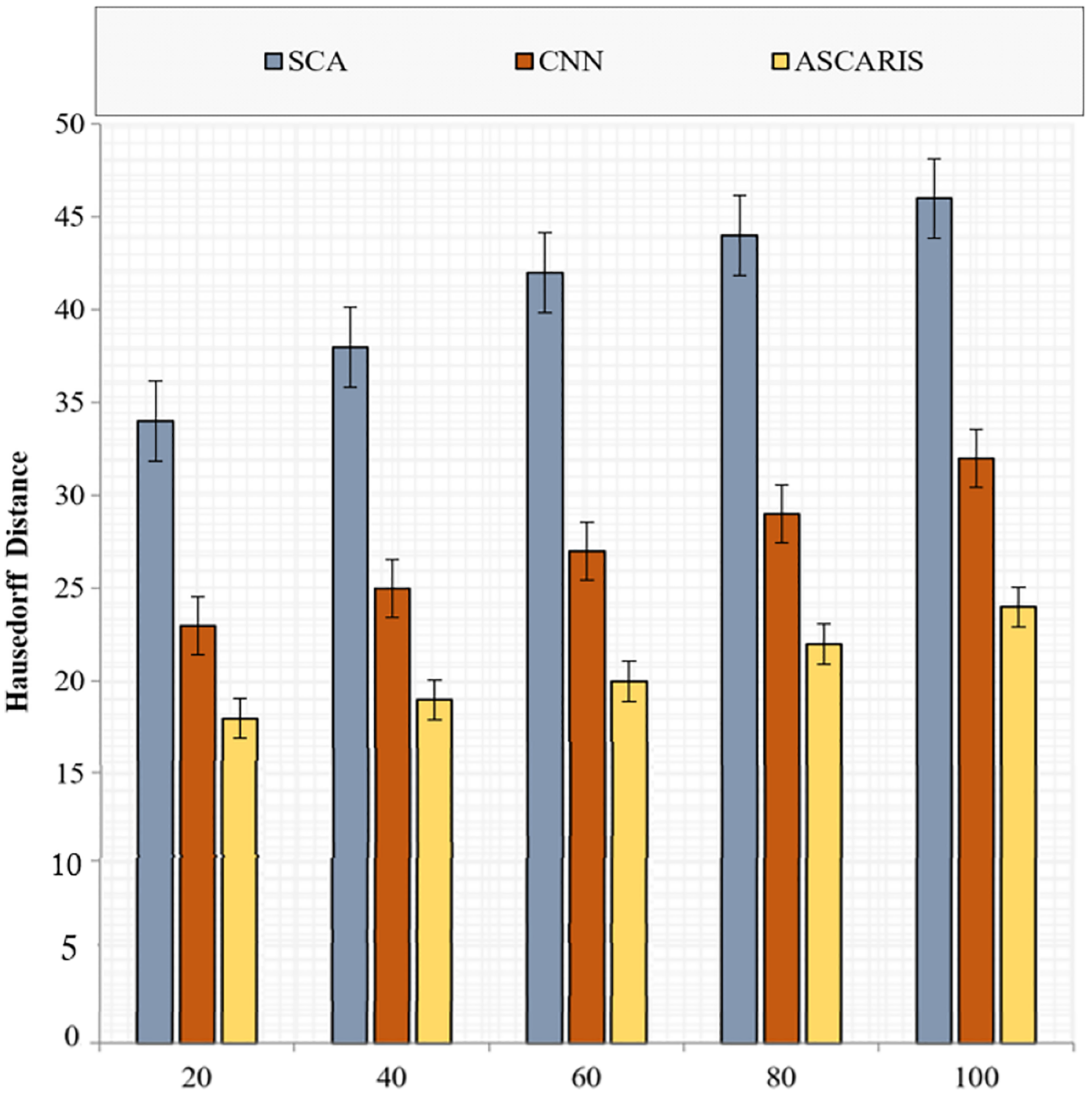
Hausedorff distance *vs* # of images.

Figure 17 illustrates the comparison of the Hausdorff distance of our proposed ASCARIS model with the existing approaches with respect to the number of images. The Hausdorff distance of the proposed approach was low due to the segmentation of preprocessed images based on the angles of the arteries. The attention estimator was also deployed to achieve better segmentation by concentrating on the important features. The existing approaches possessed increased Hausdorff distance due to the lack of consideration of the artery angle for segmentation.

##### PSNR

**Figure 18:**
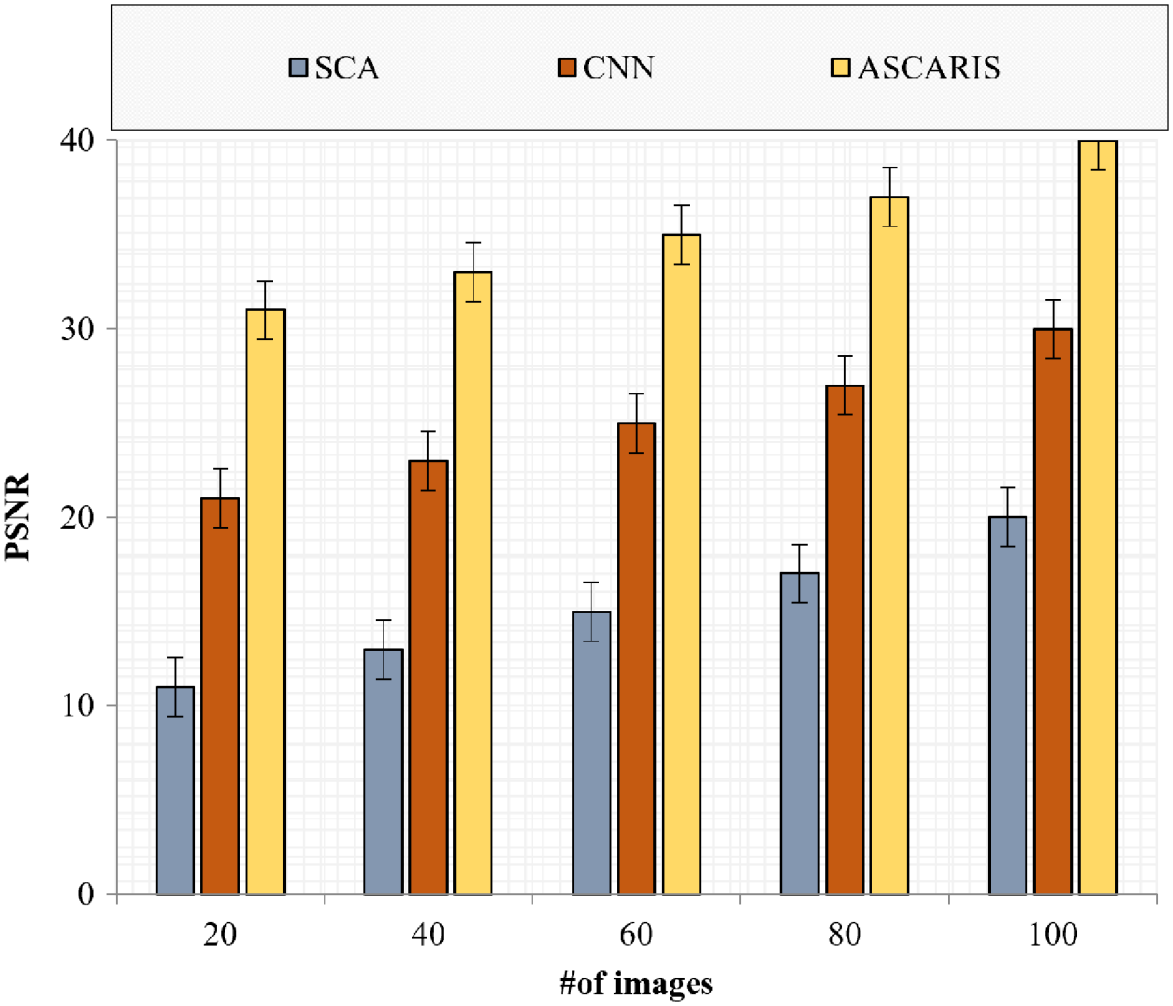
PSNR *vs* # of images.

The peak signal-to-noise ratio is a significant metric for assessing the quality of an image. The greater the value of the PSNR, the greater the quality of the image. Figure 18 denotes the comparison of the PSNR of the proposed approach with the existing approaches with respect to the number of images. The PSNR of the proposed approach was high at 35.2 due to the effective preprocessing of images. The preprocessing of images removed noise by implementing a modified Wiener filter. The contrast of the image was enhanced without affecting the edge information, resulting in an increased PSNR value. The existing approaches did not consider an effective noise removal technique, which degraded the PSNR value.

##### Segmentation accuracy

**Figure 19:**
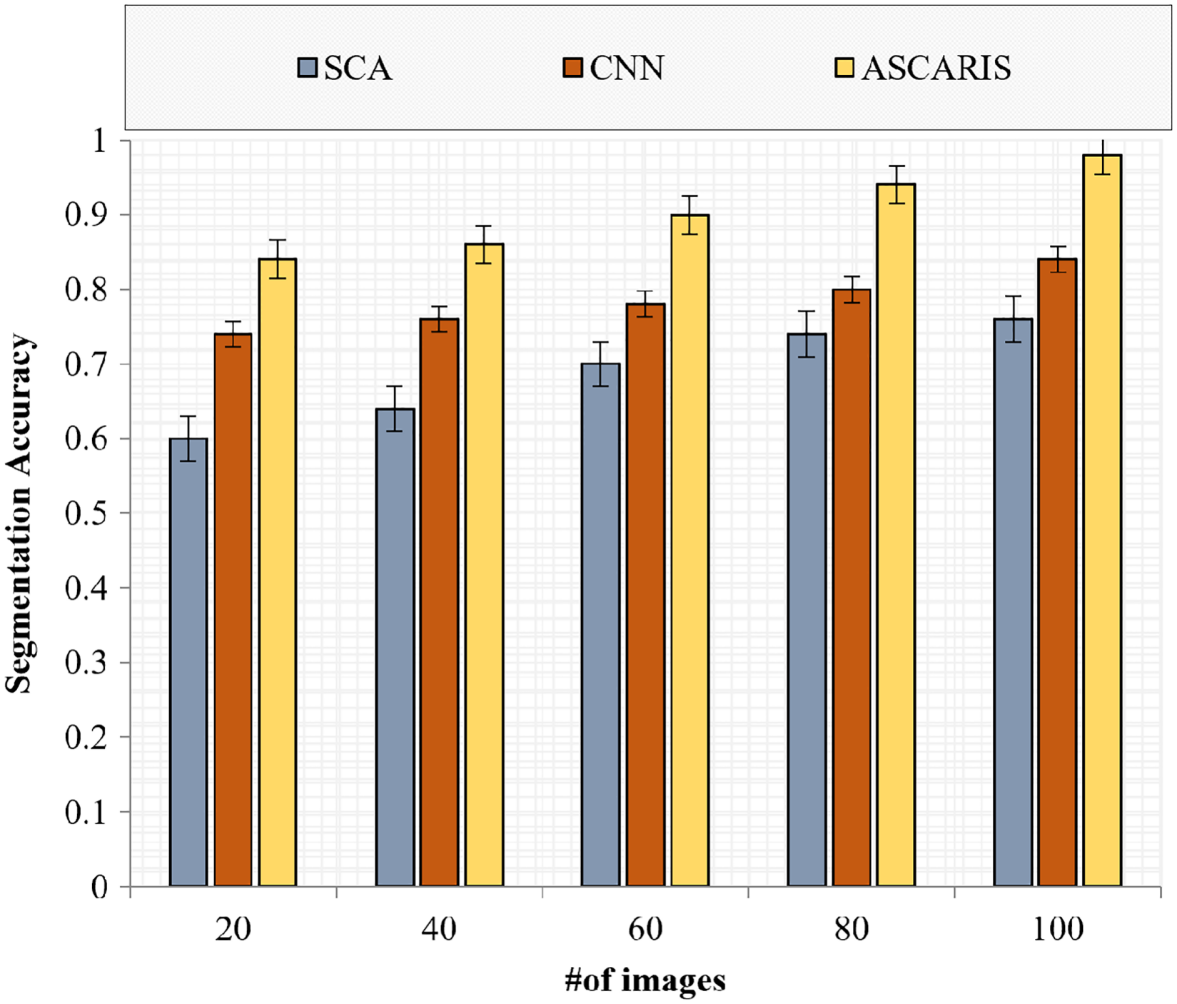
Segmentation accuracy *vs* # of images.

Segmentation accuracy (SA) is an effective metric for evaluating the accuracy of a segmentation process. The comparison of the segmentation accuracy of the proposed ASCARIS model with the existing approaches with respect to the number of images is presented in Fig. 19. The proposed approach had increased segmentation accuracy due to the implementation of the attention estimator, which was used to extract important features to perform accurate segmentation. The increased values of Jaccard similarity and Hausdorff distance achieved by the proposed approach resulted in the increased accuracy of segmentation. The existing approaches possessed less Jaccard similarity and Hausdorff distance, resulting in the reduced accuracy of similarity.

##### ROC curve

**Figure 20:**
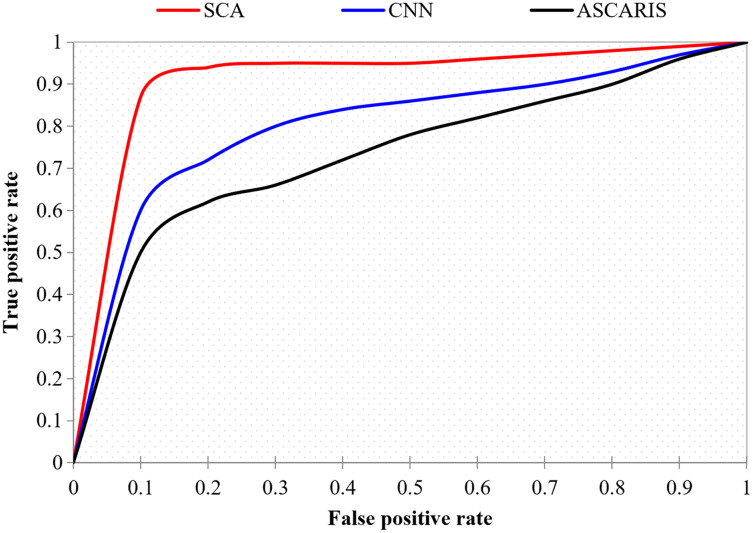
True positive rate *vs* false positive rate.

The ROC curve, also known as the receiver operating characteristic curve, is defined as a curve between the true positive and false positive of a classification approach. The ROC curve is significant in determining the accuracy of a classification approach. This metric finds the boundary between specificity and sensitivity in the classification. Figure 20 illustrates the comparison of the ROC curve of our proposed approach with the existing approaches. The proposed ASCARIS model had a steep ROC curve, which denoted the higher classification accuracy of the proposed approach than the existing approaches.

## Discussion

This section explains how the proposed ASCARIS model had improved performance compared with the previous works. In this article, the authors described the performance of the proposed work in terms of accuracy, sensitivity, specificity, ROC curve, revised contrast-to-noise ratio, dice coefficient, mean square error, Jaccard similarity, Hausdorff distance, PSNR, and segmentation accuracy with the use of a deep learning algorithm. The research outcomes were as follows:

- We presented multi-constraint-based preprocessing methods to improve the quality of the input image, and noise was removed from the X-ray angiogram image using a modified Wiener filter.
- The segmentation accuracy was very high, since the attention-based nested U-net accurately segmented the coronary artery areas and improved the edge boundaries of the image pixels.
- The proposed VGG16 architecture fixed the disadvantages of traditional CNN and machine learning. It improved the accuracy and speed (fast training and testing). The training process could be updated once for all network layers, and it did not require disk storage for feature caching.
- Our proposed segmentation and classification algorithms reduced the false positive rate, and the performance of the segmentation and classification processes improved.

The vessel segmentation method was proposed for vessel detection and segmentation in angiographic images (Xia et al., 2020). The proposed method removed the background artifacts and obtained a better vascular structure. The feature extraction phase and binarization contributed to the reduction of computational complexity.

- Background structure removal maximized segmentation accuracy and minimized the error rate, but the computational complexity in the matrix decomposition model was very high and increased the vessel segmentation time.
- Preprocessing was not concentrated; however, the X-ray angiogram images exhibited low contrast, illumination, and occlusion in the images.
- CAD prediction is implemented for early analysis of human health (Wu et al., 2020). The differentiation between the vascular structure and non-vascular structure can become tedious due to inhomogeneous intensities.
- Due to the use of deep CNN, the false positive rate was still higher. Additionally, image noise was not reduced and directly forwarded to the classifier. Furthermore, CNN did not focus on the standard orientation and position of the coronary artery. If the extracted coronary artery structure is different from the trained set, recognition accuracy is greatly affected.
- The patients’ health can vary, and deep CNN was not sufficient to adaptively work for different CAD and non-CAD patients. Furthermore, it was not suitable for large-scale datasets. The runtime for convolution operations was computationally expensive and consumed more processing time.

The coronary artery must be identified in angiograms for the timely detection and treatment of any disease of the coronary artery (Xiao et al., 2020). This article addressed the coronary vessels’ ambiguity and projected angle differences in a sequence of angiogram images.

- This work was time-consuming, since it was not possible to draw a centerline for all the complex vessels in the coronary artery image. However, the difference in shape was not focused, and it was also not suitable when the image consists of a huge number of coronary branches.
- The continuity between both open and closed curves was very hard to predict. Thus, the recognition accuracy of the coronary artery was less. In particular, the identification degree of every branch in image 1 and image 2 was required for comparison. Therefore, it did not support a large volume of the clinical database.

**Figure 21:**
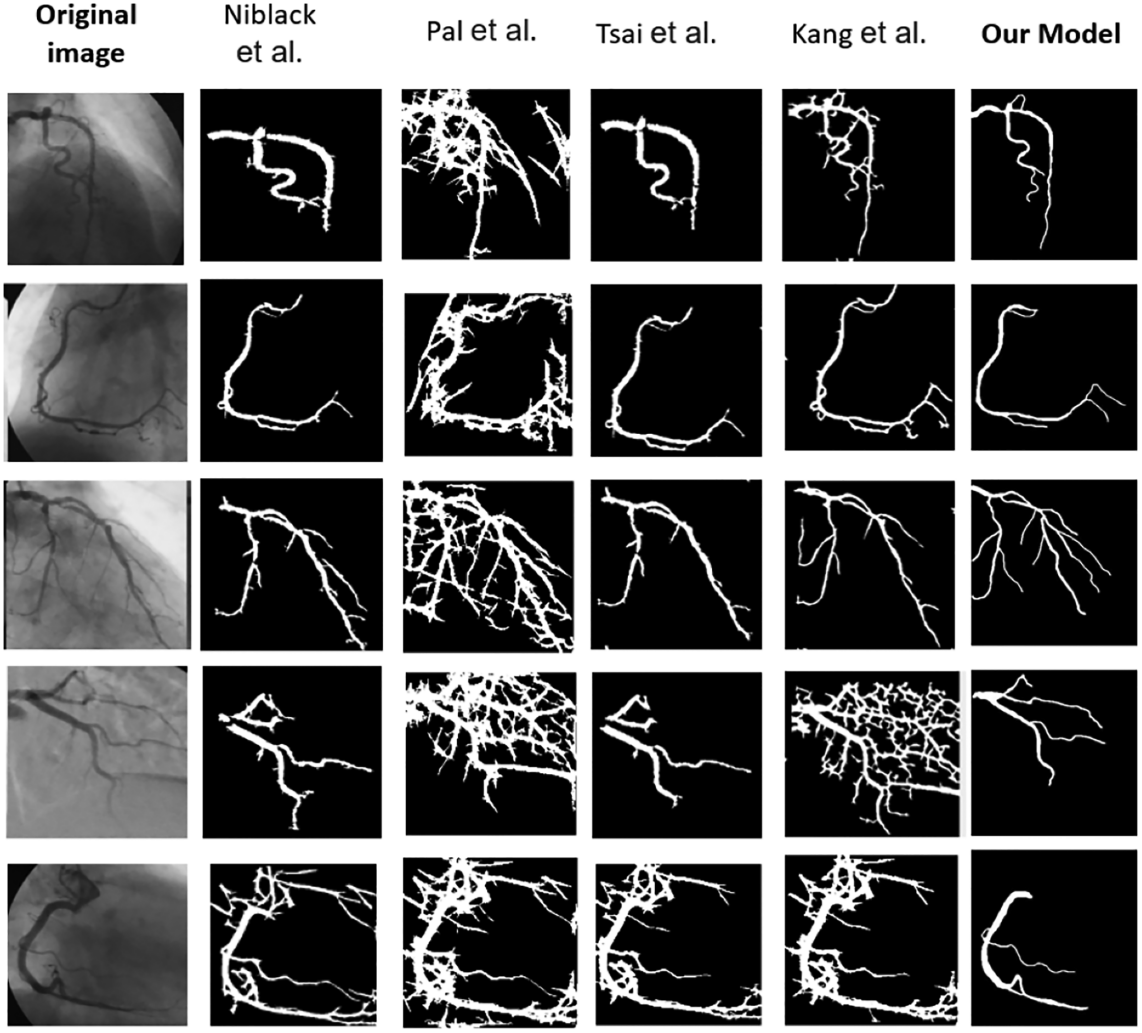
Comparison of several original and (ground truth) angiograms from the test group. For each of the studies: the method of Niblack (Niblack, 1985), the Local entropy method (Pal & Pal, 1989), the Moment-preserving method (Tsai, 1985), the Degree-based method (Kang et al., 2013) and our model.

Table 6 explains the comparison of the proposed and existing approaches by various performance metrics. It shows the average value of the performance metrics. It reveals that our proposed ASCARIS model achieved higher performance compared with existing works. Figure 21 shows the comparison of several original and (ground truth) angiograms from the test group. For each of the studies: the method of Niblack (Niblack, 1985), the Local entropy method (Pal & Pal, 1989), the Moment-preserving method (Tsai, 1985), the Degree-based method (Kang et al., 2013) and our model.

**Table 6:** Comparison of proposed and existing approaches.

Performance metrics	Proposed *vs* existing approaches		
	SCA	CNN	ASCARIS
Accuracy	0.806 $\pm \; 0.03$	0.9 $\pm \; 0.02$	0.97 $\pm \; 0.01$
Specificity	0.73 $\pm \; 0.03$	0.084 $\pm \; 0.02$	0.93 $\pm \; 0.01$
Sensitivity	0.69 $\pm \; 0.4$	0.79 $\pm \; 0.3$	0.892 $\pm \; 0.2$
rCNR	0.76 $\pm \; 0.03$	1.28 $\pm \; 0.02$	1.7 $\pm \; 0.02$
MSE	53.4 $\pm \; 0.3$	46 $\pm \; 0.2$	27.2 $\pm \; 0.1$
$DC$	0.736 $\pm \; 0.04$	0.834 $\pm \; 0.02$	0.916 $\pm \; 0.01$
$JS$	0.714 $\pm \; 0.03$	0.79 $\pm \; 0.02$	0.89 $\pm \; 0.01$
$Di{s_H}$	40.8 $\pm \; 0.4$	27.2 $\pm \; 0.3$	20.6 $\pm \; 0.2$
PSNR	15.2 $\pm \; 0.03$	25.2 $\pm \; 0.04$	35.2 $\pm \; 0.02$
SA	0.688 $\pm \; 0.04$	0.784 $\pm \; 0.02$	0.904 $\pm \; 0.01$

## Conclusions

The presence of noise in X-ray angiography and uncertainties caused by variations in angles and respiratory motion affect the detection of CAD from X-ray images. The proposed ASCARIS model overcame these limitations by performing several significant processes. Initially, the preprocessing of the input images was carried out, in which the removal of noise and the enhancement of contrast were performed. The binarization of the enhanced images was executed by implementing Otsu thresholding. The segmentation of the preprocessed images was performed by implementing an attention-based nested U-net in which the attention estimator was incorporated to overcome the difficulties in segmenting overlapped and intersecting arteries. The estimation of the artery angle further contributed to an increased segmentation accuracy of 99%. Finally, the extraction of threefold features and classification of segmented images were performed by implementing the VGG16 architecture. The experimentation of the ASCARIS model was carried out in the MATLAB R2020a simulation tool, and the validation of the proposed model was executed by comparing it with existing approaches in terms of performance metrics, such as accuracy, sensitivity, specificity, revised contrast-to-noise ratio, mean square error, dice coefficient, Jaccard similarity, Hausdorff distance, PSNR, segmentation accuracy, and ROC curve. From the results obtained, we can conclude that our proposed model outperformed the existing approaches in all metrics in the classification of CAD.

In the future, the proposed ASCARIS model will be investigated by including other datasets, and other deep learning methods may be investigated to enhance classification performance. Furthermore, the severity of the disease will also be determined to assist the diagnosis of coronary artery disease.

## Supplemental Information

10.7717/peerj-cs.993/supp-1 Supplemental Information 1 Dataset.

10.7717/peerj-cs.993/supp-2 Supplemental Information 2 Codes.

## Additional Information and Declarations

### Data Availability

The following information was supplied regarding data availability: The images of the coronary artery disease diagnostic dataset from the DCA1 and the complete implementation code and the final results in the form of images are available in the Supplemental Files. The third-party database of X-ray coronary angiograms (DCA1) is free for research and academic purposes and is available from: Cervantes-Sanchez, Fernando; Cruz-Aceves, Ivan; Hernandez-Aguirre, Arturo; Hernandez-Gonzalez, Martha A.; Solorio-Meza, Sergio E. 2019. “Automatic Segmentation of Coronary Arteries in X-ray Angiograms using Multiscale Analysis and Artificial Neural Networks.” Applied Sciences 9, no. 24: 5507. https://doi.org/10.3390/app9245507; http://personal.cimat.mx:8181/~ivan.cruz/DB_Angiograms.html.
