# Supplementary figures and images for: Multi-constraints based deep learning model for automated segmentation and diagnosis of coronary artery disease in X-ray angiographic images (part 2 of 2)
Source: PeerJ Comput Sci. 2022 Jun 3;8:e993. doi: 10.7717/peerj-cs.993 (PMC9202622; doi:10.7717/peerj-cs.993)

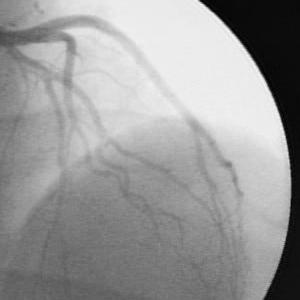

Supplement: Supplemental Information 1 [file peerj-cs-08-993-s001.zip › dataset files/Dataset/69.jpg]

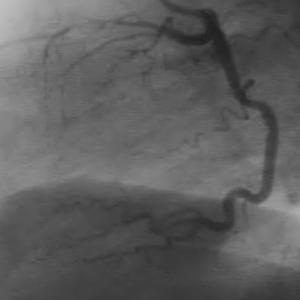

Supplement: Supplemental Information 1 [file peerj-cs-08-993-s001.zip › dataset files/Dataset/7.jpg]

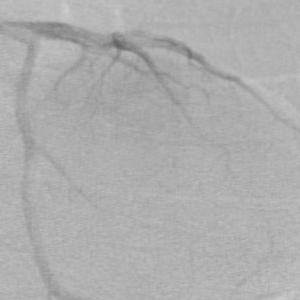

Supplement: Supplemental Information 1 [file peerj-cs-08-993-s001.zip › dataset files/Dataset/70.jpg]

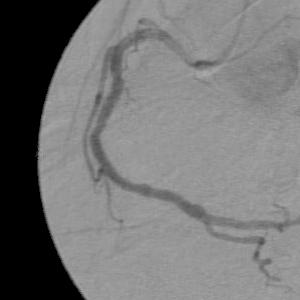

Supplement: Supplemental Information 1 [file peerj-cs-08-993-s001.zip › dataset files/Dataset/71.jpg]

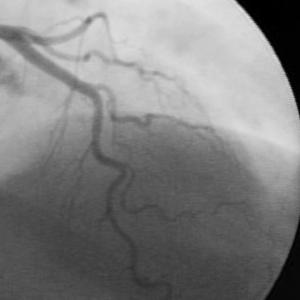

Supplement: Supplemental Information 1 [file peerj-cs-08-993-s001.zip › dataset files/Dataset/72.jpg]

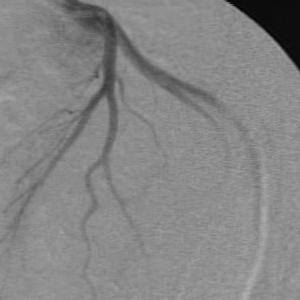

Supplement: Supplemental Information 1 [file peerj-cs-08-993-s001.zip › dataset files/Dataset/73.jpg]

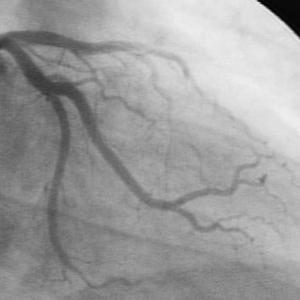

Supplement: Supplemental Information 1 [file peerj-cs-08-993-s001.zip › dataset files/Dataset/74.jpg]

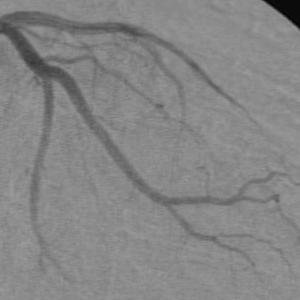

Supplement: Supplemental Information 1 [file peerj-cs-08-993-s001.zip › dataset files/Dataset/75.jpg]

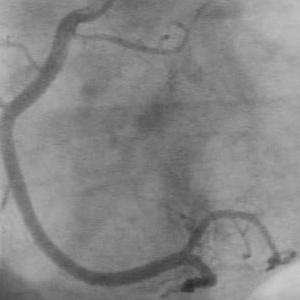

Supplement: Supplemental Information 1 [file peerj-cs-08-993-s001.zip › dataset files/Dataset/76.jpg]

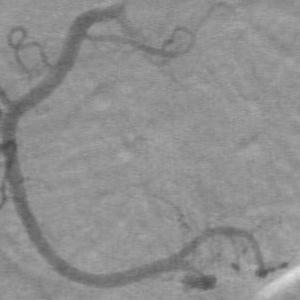

Supplement: Supplemental Information 1 [file peerj-cs-08-993-s001.zip › dataset files/Dataset/77.jpg]

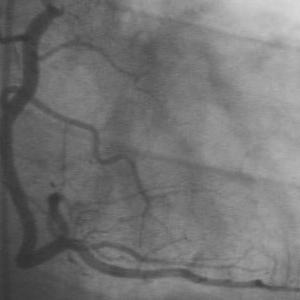

Supplement: Supplemental Information 1 [file peerj-cs-08-993-s001.zip › dataset files/Dataset/78.jpg]

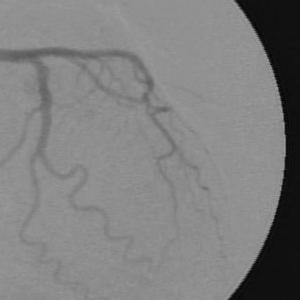

Supplement: Supplemental Information 1 [file peerj-cs-08-993-s001.zip › dataset files/Dataset/79.jpg]

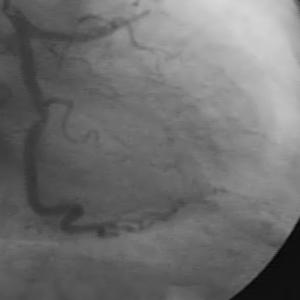

Supplement: Supplemental Information 1 [file peerj-cs-08-993-s001.zip › dataset files/Dataset/8.jpg]

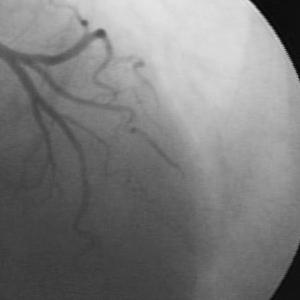

Supplement: Supplemental Information 1 [file peerj-cs-08-993-s001.zip › dataset files/Dataset/80.jpg]

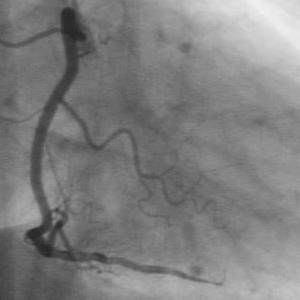

Supplement: Supplemental Information 1 [file peerj-cs-08-993-s001.zip › dataset files/Dataset/81.jpg]

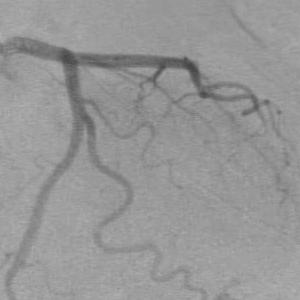

Supplement: Supplemental Information 1 [file peerj-cs-08-993-s001.zip › dataset files/Dataset/82.jpg]

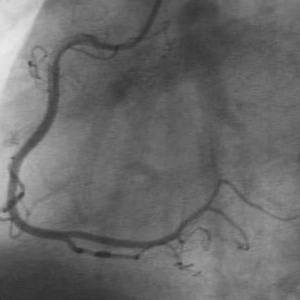

Supplement: Supplemental Information 1 [file peerj-cs-08-993-s001.zip › dataset files/Dataset/83.jpg]

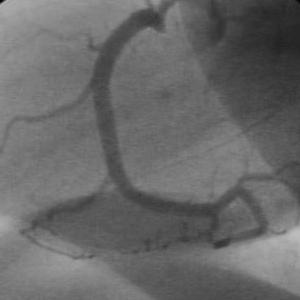

Supplement: Supplemental Information 1 [file peerj-cs-08-993-s001.zip › dataset files/Dataset/84.jpg]

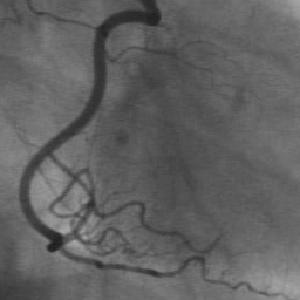

Supplement: Supplemental Information 1 [file peerj-cs-08-993-s001.zip › dataset files/Dataset/85.jpg]

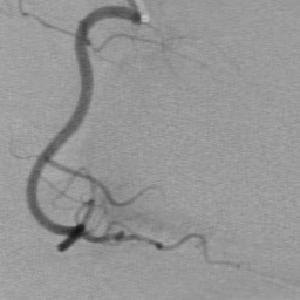

Supplement: Supplemental Information 1 [file peerj-cs-08-993-s001.zip › dataset files/Dataset/86.jpg]

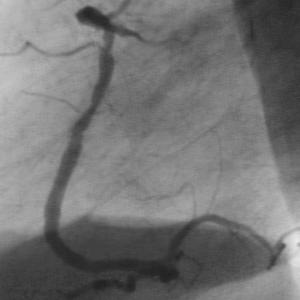

Supplement: Supplemental Information 1 [file peerj-cs-08-993-s001.zip › dataset files/Dataset/87.jpg]

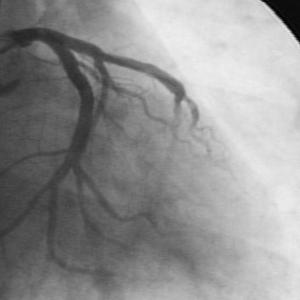

Supplement: Supplemental Information 1 [file peerj-cs-08-993-s001.zip › dataset files/Dataset/88.jpg]

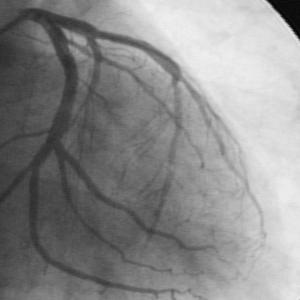

Supplement: Supplemental Information 1 [file peerj-cs-08-993-s001.zip › dataset files/Dataset/89.jpg]

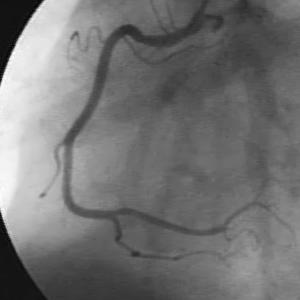

Supplement: Supplemental Information 1 [file peerj-cs-08-993-s001.zip › dataset files/Dataset/9.jpg]

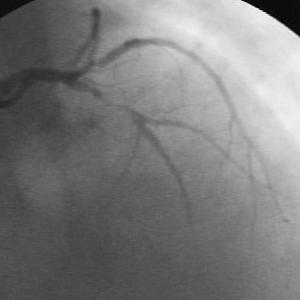

Supplement: Supplemental Information 1 [file peerj-cs-08-993-s001.zip › dataset files/Dataset/90.jpg]

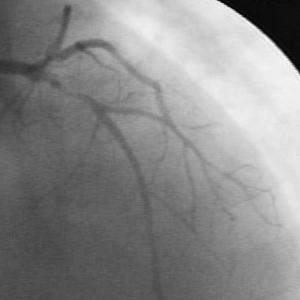

Supplement: Supplemental Information 1 [file peerj-cs-08-993-s001.zip › dataset files/Dataset/91.jpg]

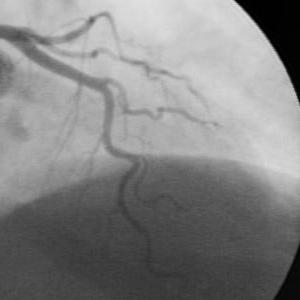

Supplement: Supplemental Information 1 [file peerj-cs-08-993-s001.zip › dataset files/Dataset/92.jpg]

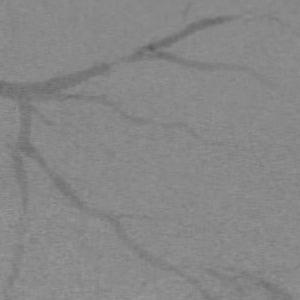

Supplement: Supplemental Information 1 [file peerj-cs-08-993-s001.zip › dataset files/Dataset/93.jpg]

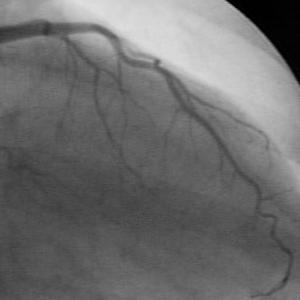

Supplement: Supplemental Information 1 [file peerj-cs-08-993-s001.zip › dataset files/Dataset/94.jpg]

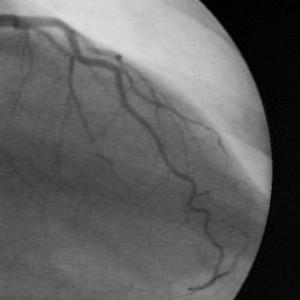

Supplement: Supplemental Information 1 [file peerj-cs-08-993-s001.zip › dataset files/Dataset/95.jpg]

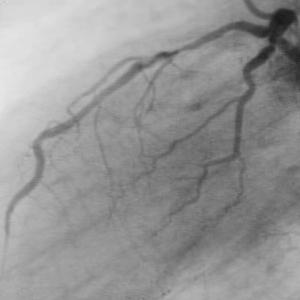

Supplement: Supplemental Information 1 [file peerj-cs-08-993-s001.zip › dataset files/Dataset/96.jpg]

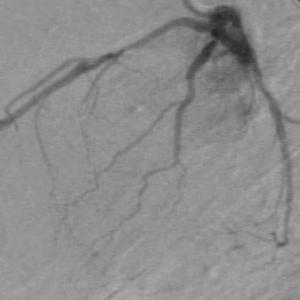

Supplement: Supplemental Information 1 [file peerj-cs-08-993-s001.zip › dataset files/Dataset/97.jpg]

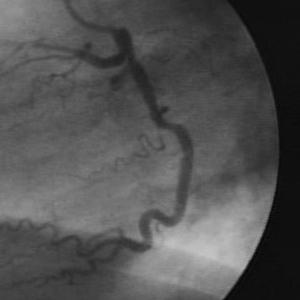

Supplement: Supplemental Information 1 [file peerj-cs-08-993-s001.zip › dataset files/Dataset/98.jpg]

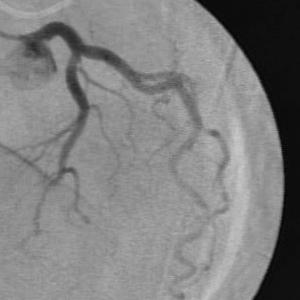

Supplement: Supplemental Information 1 [file peerj-cs-08-993-s001.zip › dataset files/Dataset/99.jpg]

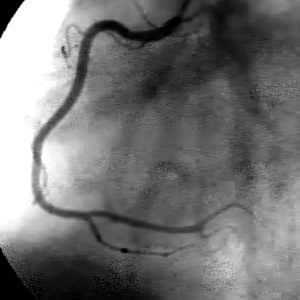

Supplement: Supplemental Information 2 [file peerj-cs-08-993-s002.zip › code files/Existing/ContrastFilledRes.jpg]

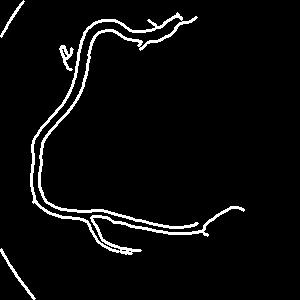

Supplement: Supplemental Information 2 [file peerj-cs-08-993-s002.zip › code files/Existing/segRes.jpg]

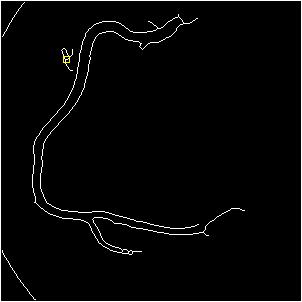

Supplement: Supplemental Information 2 [file peerj-cs-08-993-s002.zip › code files/Existing/SeqFpsRes.jpg]

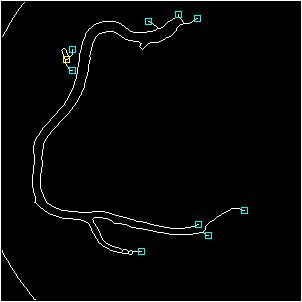

Supplement: Supplemental Information 2 [file peerj-cs-08-993-s002.zip › code files/Existing/StenosisDetRes.jpg]

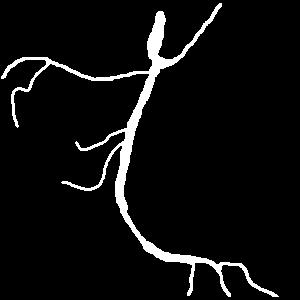

Supplement: Supplemental Information 2 [file peerj-cs-08-993-s002.zip › code files/Existing/GroundTruth/100_gt.jpg]

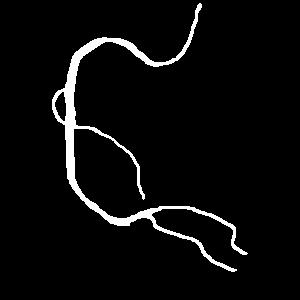

Supplement: Supplemental Information 2 [file peerj-cs-08-993-s002.zip › code files/Existing/GroundTruth/101_gt.jpg]

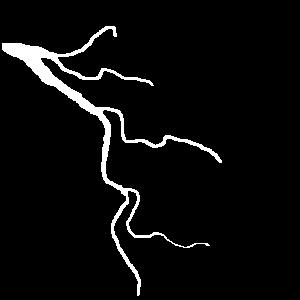

Supplement: Supplemental Information 2 [file peerj-cs-08-993-s002.zip › code files/Existing/GroundTruth/102_gt.jpg]

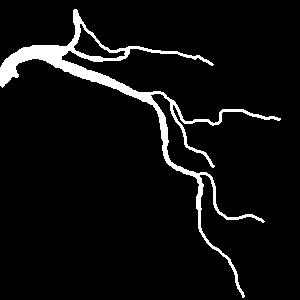

Supplement: Supplemental Information 2 [file peerj-cs-08-993-s002.zip › code files/Existing/GroundTruth/103_gt.jpg]

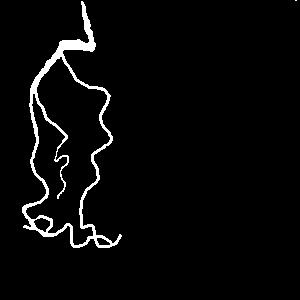

Supplement: Supplemental Information 2 [file peerj-cs-08-993-s002.zip › code files/Existing/GroundTruth/104_gt.jpg]

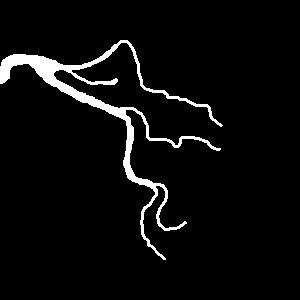

Supplement: Supplemental Information 2 [file peerj-cs-08-993-s002.zip › code files/Existing/GroundTruth/105_gt.jpg]

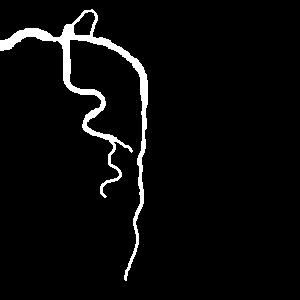

Supplement: Supplemental Information 2 [file peerj-cs-08-993-s002.zip › code files/Existing/GroundTruth/106_gt.jpg]

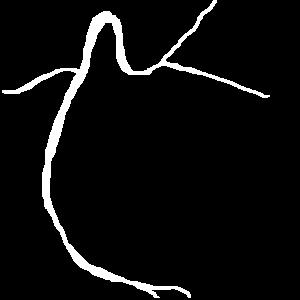

Supplement: Supplemental Information 2 [file peerj-cs-08-993-s002.zip › code files/Existing/GroundTruth/107_gt.jpg]

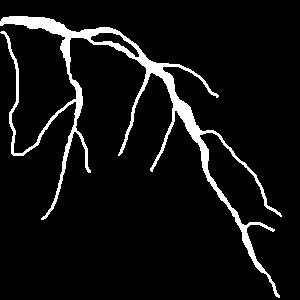

Supplement: Supplemental Information 2 [file peerj-cs-08-993-s002.zip › code files/Existing/GroundTruth/108_gt.jpg]

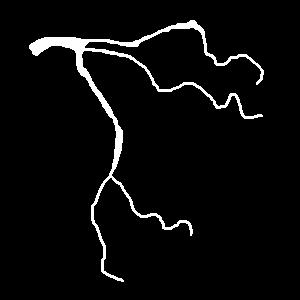

Supplement: Supplemental Information 2 [file peerj-cs-08-993-s002.zip › code files/Existing/GroundTruth/109_gt.jpg]

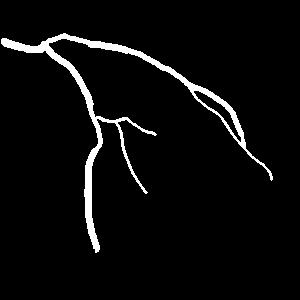

Supplement: Supplemental Information 2 [file peerj-cs-08-993-s002.zip › code files/Existing/GroundTruth/10_gt.jpg]

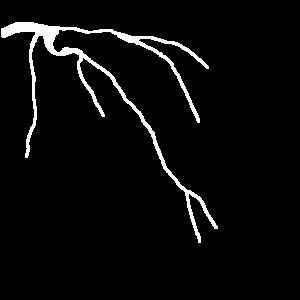

Supplement: Supplemental Information 2 [file peerj-cs-08-993-s002.zip › code files/Existing/GroundTruth/110_gt.jpg]

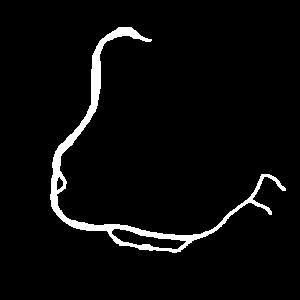

Supplement: Supplemental Information 2 [file peerj-cs-08-993-s002.zip › code files/Existing/GroundTruth/111_gt.jpg]

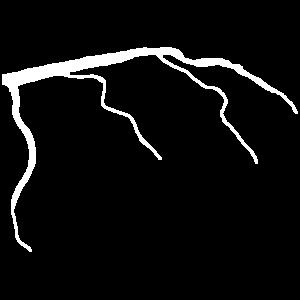

Supplement: Supplemental Information 2 [file peerj-cs-08-993-s002.zip › code files/Existing/GroundTruth/112_gt.jpg]

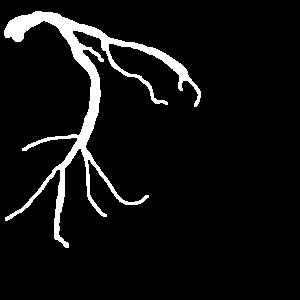

Supplement: Supplemental Information 2 [file peerj-cs-08-993-s002.zip › code files/Existing/GroundTruth/113_gt.jpg]

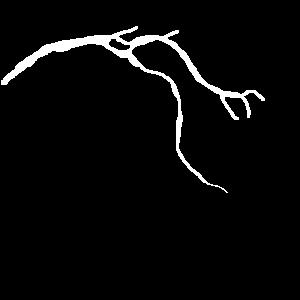

Supplement: Supplemental Information 2 [file peerj-cs-08-993-s002.zip › code files/Existing/GroundTruth/114_gt.jpg]

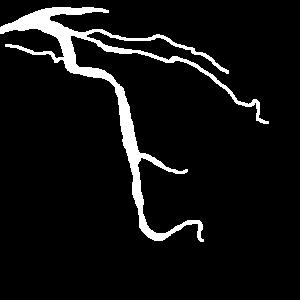

Supplement: Supplemental Information 2 [file peerj-cs-08-993-s002.zip › code files/Existing/GroundTruth/115_gt.jpg]

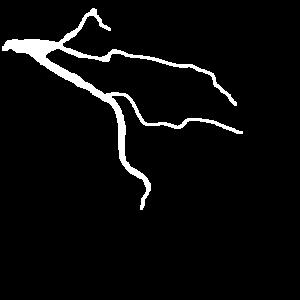

Supplement: Supplemental Information 2 [file peerj-cs-08-993-s002.zip › code files/Existing/GroundTruth/116_gt.jpg]

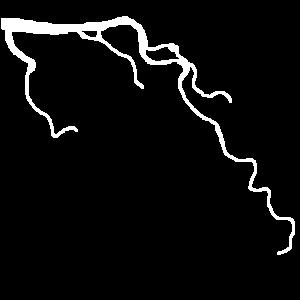

Supplement: Supplemental Information 2 [file peerj-cs-08-993-s002.zip › code files/Existing/GroundTruth/117_gt.jpg]

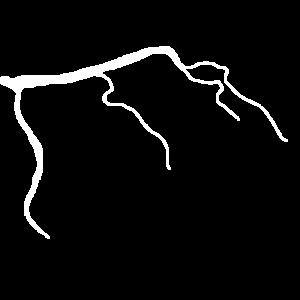

Supplement: Supplemental Information 2 [file peerj-cs-08-993-s002.zip › code files/Existing/GroundTruth/118_gt.jpg]

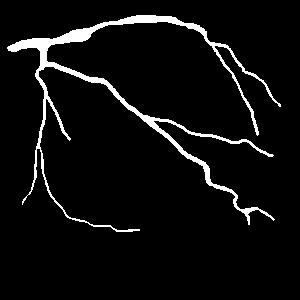

Supplement: Supplemental Information 2 [file peerj-cs-08-993-s002.zip › code files/Existing/GroundTruth/119_gt.jpg]

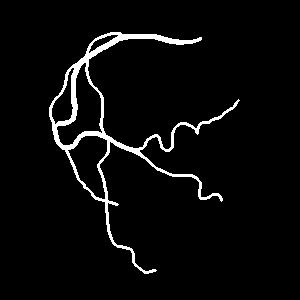

Supplement: Supplemental Information 2 [file peerj-cs-08-993-s002.zip › code files/Existing/GroundTruth/11_gt.jpg]

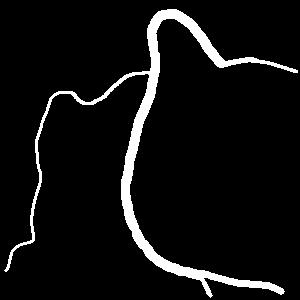

Supplement: Supplemental Information 2 [file peerj-cs-08-993-s002.zip › code files/Existing/GroundTruth/120_gt.jpg]

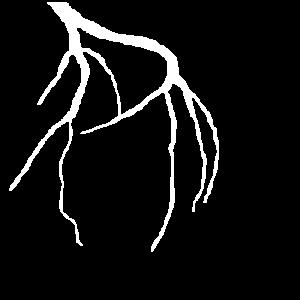

Supplement: Supplemental Information 2 [file peerj-cs-08-993-s002.zip › code files/Existing/GroundTruth/121_gt.jpg]

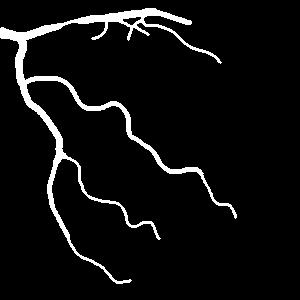

Supplement: Supplemental Information 2 [file peerj-cs-08-993-s002.zip › code files/Existing/GroundTruth/122_gt.jpg]

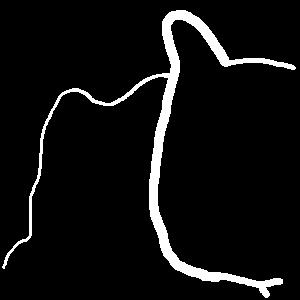

Supplement: Supplemental Information 2 [file peerj-cs-08-993-s002.zip › code files/Existing/GroundTruth/123_gt.jpg]

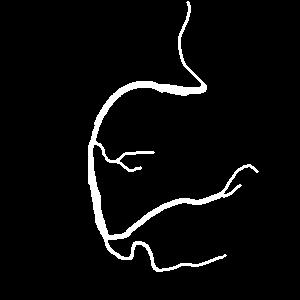

Supplement: Supplemental Information 2 [file peerj-cs-08-993-s002.zip › code files/Existing/GroundTruth/124_gt.jpg]

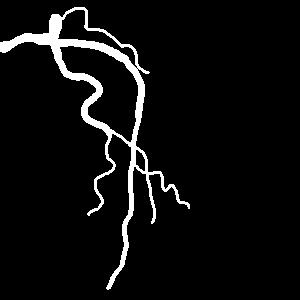

Supplement: Supplemental Information 2 [file peerj-cs-08-993-s002.zip › code files/Existing/GroundTruth/125_gt.jpg]

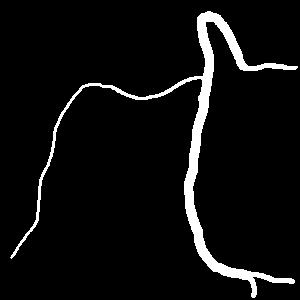

Supplement: Supplemental Information 2 [file peerj-cs-08-993-s002.zip › code files/Existing/GroundTruth/126_gt.jpg]

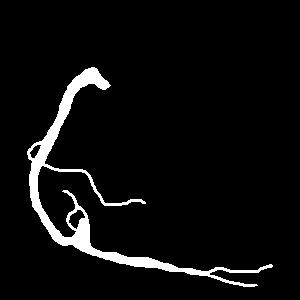

Supplement: Supplemental Information 2 [file peerj-cs-08-993-s002.zip › code files/Existing/GroundTruth/127_gt.jpg]

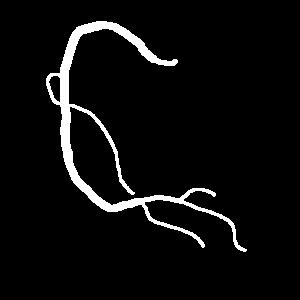

Supplement: Supplemental Information 2 [file peerj-cs-08-993-s002.zip › code files/Existing/GroundTruth/128_gt.jpg]

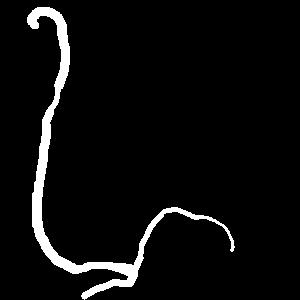

Supplement: Supplemental Information 2 [file peerj-cs-08-993-s002.zip › code files/Existing/GroundTruth/129_gt.jpg]

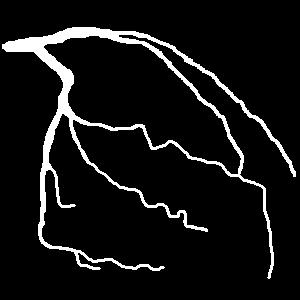

Supplement: Supplemental Information 2 [file peerj-cs-08-993-s002.zip › code files/Existing/GroundTruth/12_gt.jpg]

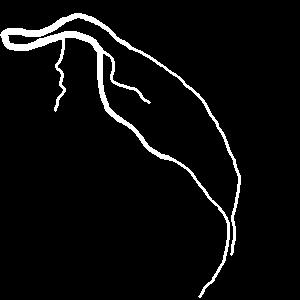

Supplement: Supplemental Information 2 [file peerj-cs-08-993-s002.zip › code files/Existing/GroundTruth/130_gt.jpg]

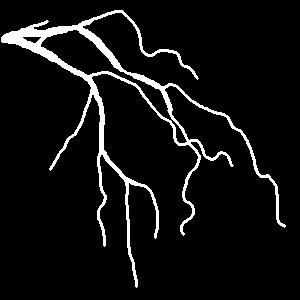

Supplement: Supplemental Information 2 [file peerj-cs-08-993-s002.zip › code files/Existing/GroundTruth/131_gt.jpg]

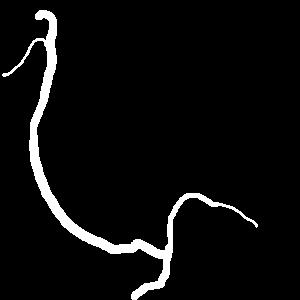

Supplement: Supplemental Information 2 [file peerj-cs-08-993-s002.zip › code files/Existing/GroundTruth/132_gt.jpg]

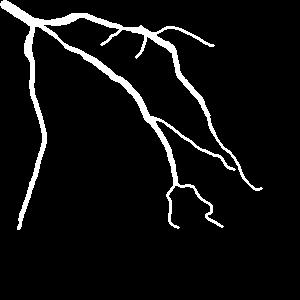

Supplement: Supplemental Information 2 [file peerj-cs-08-993-s002.zip › code files/Existing/GroundTruth/133_gt.jpg]

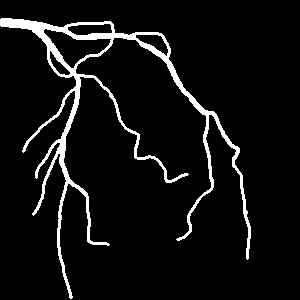

Supplement: Supplemental Information 2 [file peerj-cs-08-993-s002.zip › code files/Existing/GroundTruth/134_gt.jpg]

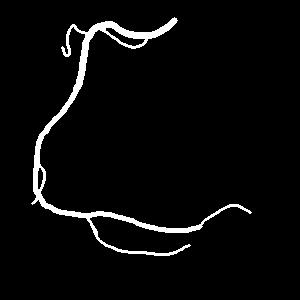

Supplement: Supplemental Information 2 [file peerj-cs-08-993-s002.zip › code files/Existing/GroundTruth/13_gt.jpg]

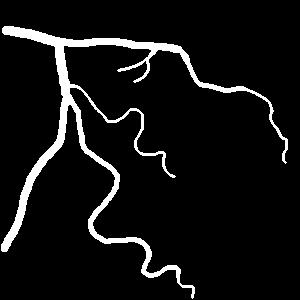

Supplement: Supplemental Information 2 [file peerj-cs-08-993-s002.zip › code files/Existing/GroundTruth/14_gt.jpg]

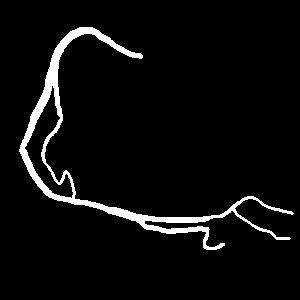

Supplement: Supplemental Information 2 [file peerj-cs-08-993-s002.zip › code files/Existing/GroundTruth/15_gt.jpg]

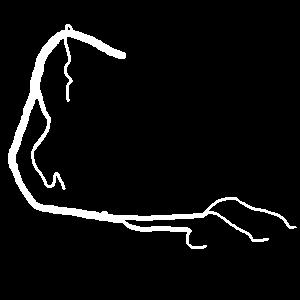

Supplement: Supplemental Information 2 [file peerj-cs-08-993-s002.zip › code files/Existing/GroundTruth/16_gt.jpg]

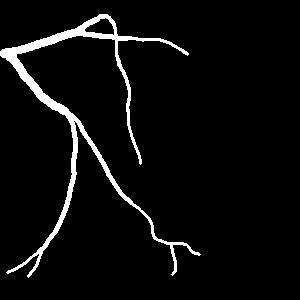

Supplement: Supplemental Information 2 [file peerj-cs-08-993-s002.zip › code files/Existing/GroundTruth/17_gt.jpg]

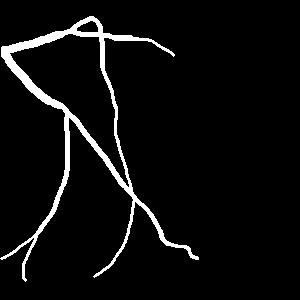

Supplement: Supplemental Information 2 [file peerj-cs-08-993-s002.zip › code files/Existing/GroundTruth/18_gt.jpg]

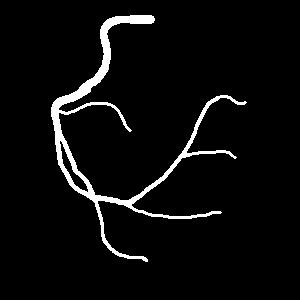

Supplement: Supplemental Information 2 [file peerj-cs-08-993-s002.zip › code files/Existing/GroundTruth/19_gt.jpg]

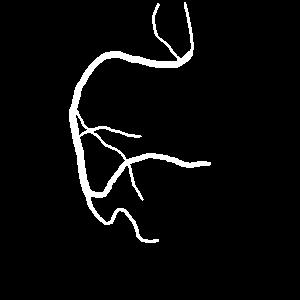

Supplement: Supplemental Information 2 [file peerj-cs-08-993-s002.zip › code files/Existing/GroundTruth/1_gt.jpg]

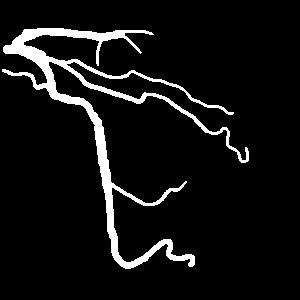

Supplement: Supplemental Information 2 [file peerj-cs-08-993-s002.zip › code files/Existing/GroundTruth/20_gt.jpg]

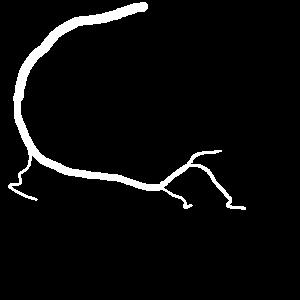

Supplement: Supplemental Information 2 [file peerj-cs-08-993-s002.zip › code files/Existing/GroundTruth/21_gt.jpg]

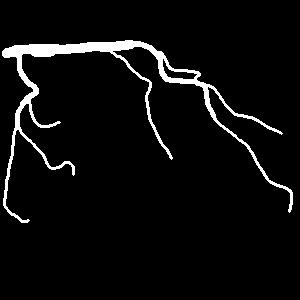

Supplement: Supplemental Information 2 [file peerj-cs-08-993-s002.zip › code files/Existing/GroundTruth/22_gt.jpg]

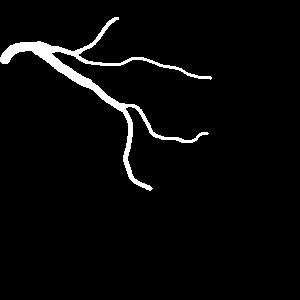

Supplement: Supplemental Information 2 [file peerj-cs-08-993-s002.zip › code files/Existing/GroundTruth/23_gt.jpg]

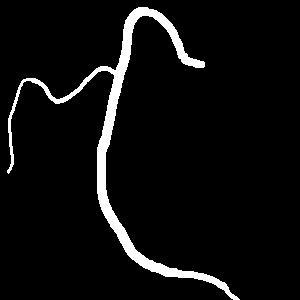

Supplement: Supplemental Information 2 [file peerj-cs-08-993-s002.zip › code files/Existing/GroundTruth/24_gt.jpg]

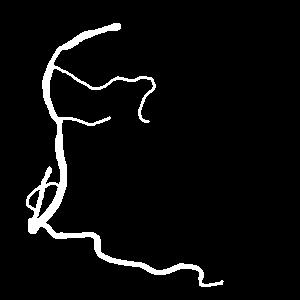

Supplement: Supplemental Information 2 [file peerj-cs-08-993-s002.zip › code files/Existing/GroundTruth/25_gt.jpg]

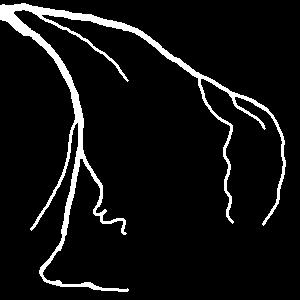

Supplement: Supplemental Information 2 [file peerj-cs-08-993-s002.zip › code files/Existing/GroundTruth/26_gt.jpg]

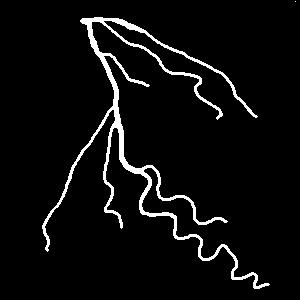

Supplement: Supplemental Information 2 [file peerj-cs-08-993-s002.zip › code files/Existing/GroundTruth/27_gt.jpg]

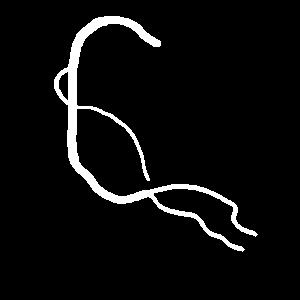

Supplement: Supplemental Information 2 [file peerj-cs-08-993-s002.zip › code files/Existing/GroundTruth/28_gt.jpg]

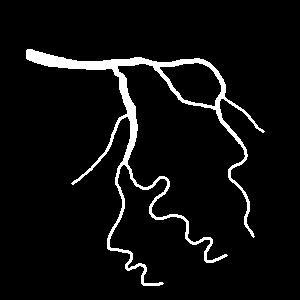

Supplement: Supplemental Information 2 [file peerj-cs-08-993-s002.zip › code files/Existing/GroundTruth/29_gt.jpg]

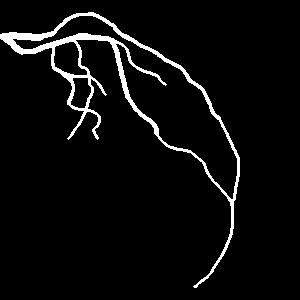

Supplement: Supplemental Information 2 [file peerj-cs-08-993-s002.zip › code files/Existing/GroundTruth/2_gt.jpg]
