# Supplementary figures and images for: Multi-constraints based deep learning model for automated segmentation and diagnosis of coronary artery disease in X-ray angiographic images (part 1 of 2)
Source: PeerJ Comput Sci. 2022 Jun 3;8:e993. doi: 10.7717/peerj-cs.993 (PMC9202622; doi:10.7717/peerj-cs.993)

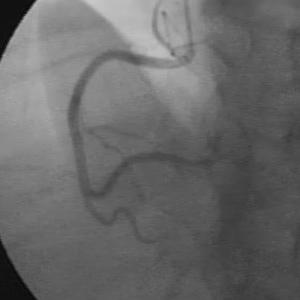

Supplement: Supplemental Information 1 [file peerj-cs-08-993-s001.zip › dataset files/Dataset/1.jpg]

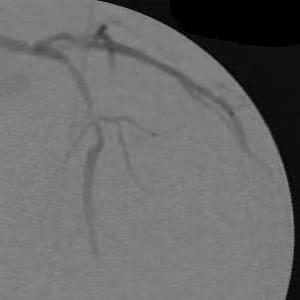

Supplement: Supplemental Information 1 [file peerj-cs-08-993-s001.zip › dataset files/Dataset/10.jpg]

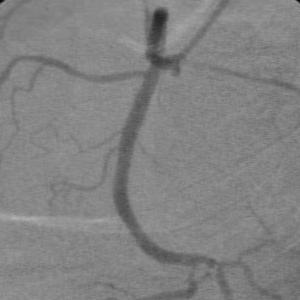

Supplement: Supplemental Information 1 [file peerj-cs-08-993-s001.zip › dataset files/Dataset/100.jpg]

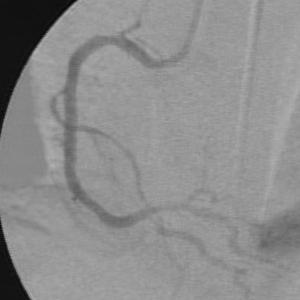

Supplement: Supplemental Information 1 [file peerj-cs-08-993-s001.zip › dataset files/Dataset/101.jpg]

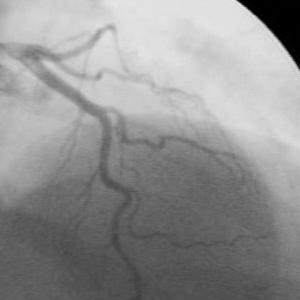

Supplement: Supplemental Information 1 [file peerj-cs-08-993-s001.zip › dataset files/Dataset/102.jpg]

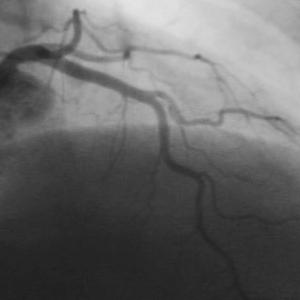

Supplement: Supplemental Information 1 [file peerj-cs-08-993-s001.zip › dataset files/Dataset/103.jpg]

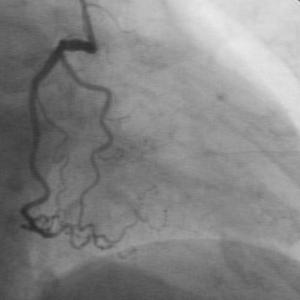

Supplement: Supplemental Information 1 [file peerj-cs-08-993-s001.zip › dataset files/Dataset/104.jpg]

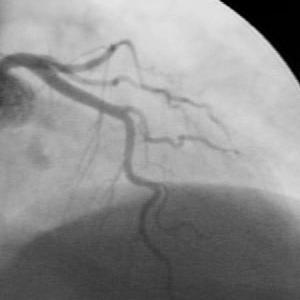

Supplement: Supplemental Information 1 [file peerj-cs-08-993-s001.zip › dataset files/Dataset/105.jpg]

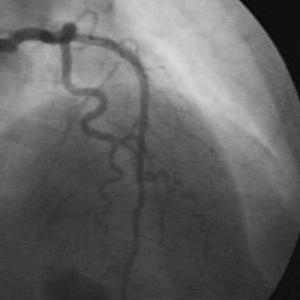

Supplement: Supplemental Information 1 [file peerj-cs-08-993-s001.zip › dataset files/Dataset/106.jpg]

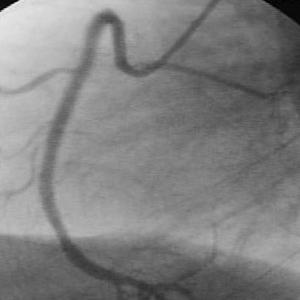

Supplement: Supplemental Information 1 [file peerj-cs-08-993-s001.zip › dataset files/Dataset/107.jpg]

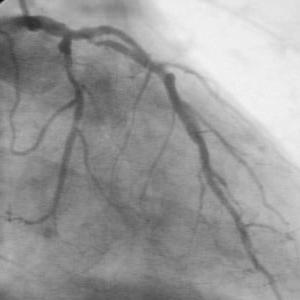

Supplement: Supplemental Information 1 [file peerj-cs-08-993-s001.zip › dataset files/Dataset/108.jpg]

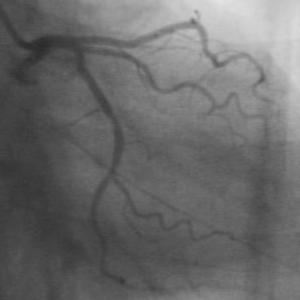

Supplement: Supplemental Information 1 [file peerj-cs-08-993-s001.zip › dataset files/Dataset/109.jpg]

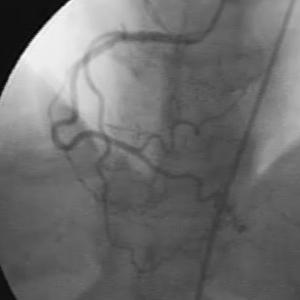

Supplement: Supplemental Information 1 [file peerj-cs-08-993-s001.zip › dataset files/Dataset/11.jpg]

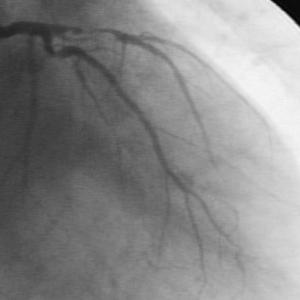

Supplement: Supplemental Information 1 [file peerj-cs-08-993-s001.zip › dataset files/Dataset/110.jpg]

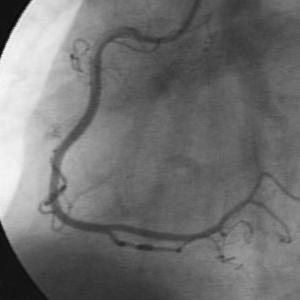

Supplement: Supplemental Information 1 [file peerj-cs-08-993-s001.zip › dataset files/Dataset/111.jpg]

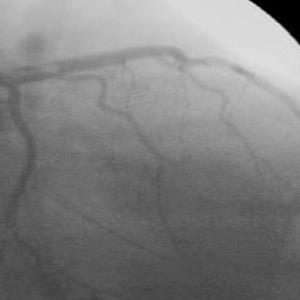

Supplement: Supplemental Information 1 [file peerj-cs-08-993-s001.zip › dataset files/Dataset/112.jpg]

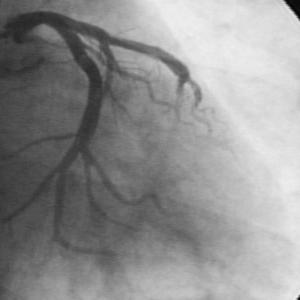

Supplement: Supplemental Information 1 [file peerj-cs-08-993-s001.zip › dataset files/Dataset/113.jpg]

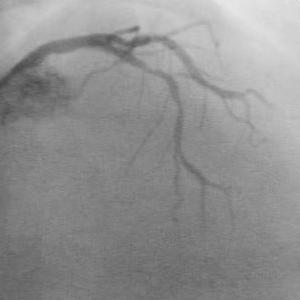

Supplement: Supplemental Information 1 [file peerj-cs-08-993-s001.zip › dataset files/Dataset/114.jpg]

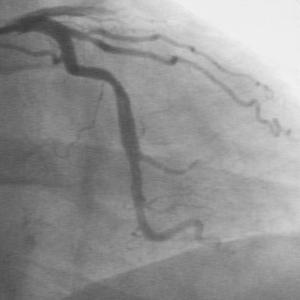

Supplement: Supplemental Information 1 [file peerj-cs-08-993-s001.zip › dataset files/Dataset/115.jpg]

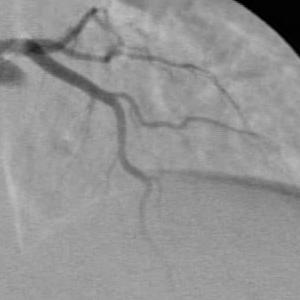

Supplement: Supplemental Information 1 [file peerj-cs-08-993-s001.zip › dataset files/Dataset/116.jpg]

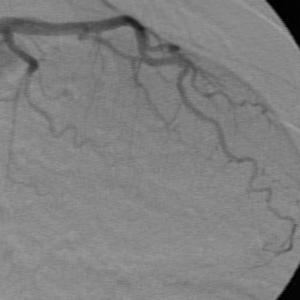

Supplement: Supplemental Information 1 [file peerj-cs-08-993-s001.zip › dataset files/Dataset/117.jpg]

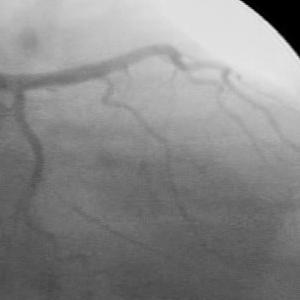

Supplement: Supplemental Information 1 [file peerj-cs-08-993-s001.zip › dataset files/Dataset/118.jpg]

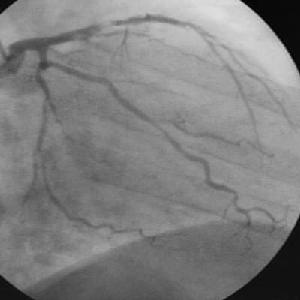

Supplement: Supplemental Information 1 [file peerj-cs-08-993-s001.zip › dataset files/Dataset/119.jpg]

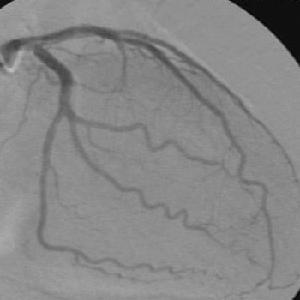

Supplement: Supplemental Information 1 [file peerj-cs-08-993-s001.zip › dataset files/Dataset/12.jpg]

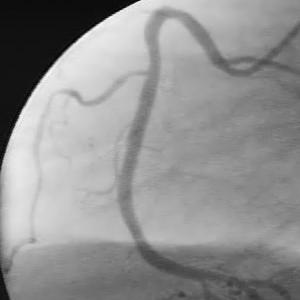

Supplement: Supplemental Information 1 [file peerj-cs-08-993-s001.zip › dataset files/Dataset/120.jpg]

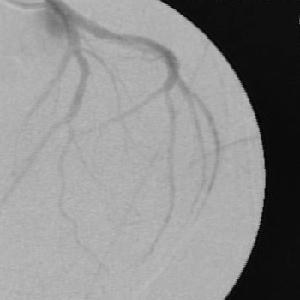

Supplement: Supplemental Information 1 [file peerj-cs-08-993-s001.zip › dataset files/Dataset/121.jpg]

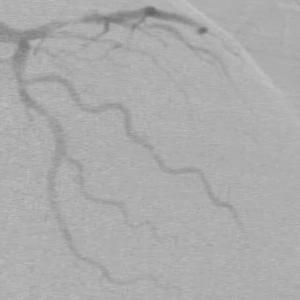

Supplement: Supplemental Information 1 [file peerj-cs-08-993-s001.zip › dataset files/Dataset/122.jpg]

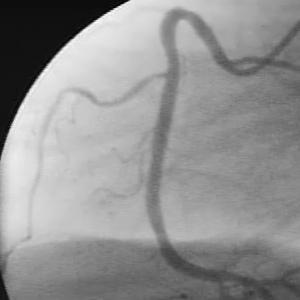

Supplement: Supplemental Information 1 [file peerj-cs-08-993-s001.zip › dataset files/Dataset/123.jpg]

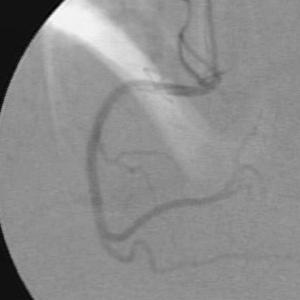

Supplement: Supplemental Information 1 [file peerj-cs-08-993-s001.zip › dataset files/Dataset/124.jpg]

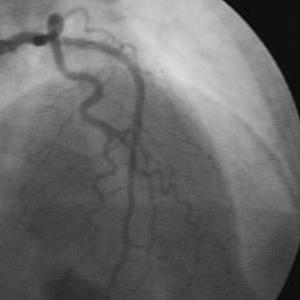

Supplement: Supplemental Information 1 [file peerj-cs-08-993-s001.zip › dataset files/Dataset/125.jpg]

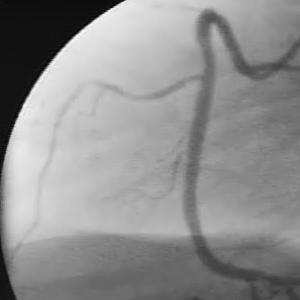

Supplement: Supplemental Information 1 [file peerj-cs-08-993-s001.zip › dataset files/Dataset/126.jpg]

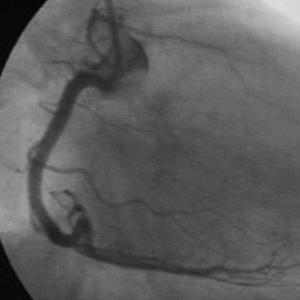

Supplement: Supplemental Information 1 [file peerj-cs-08-993-s001.zip › dataset files/Dataset/127.jpg]

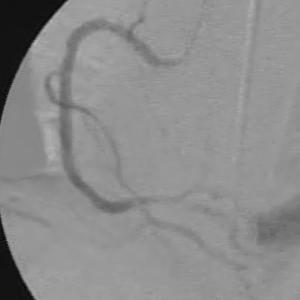

Supplement: Supplemental Information 1 [file peerj-cs-08-993-s001.zip › dataset files/Dataset/128.jpg]

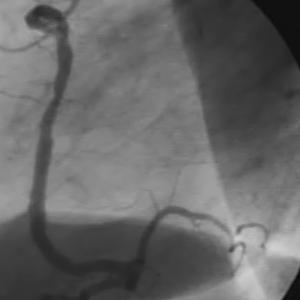

Supplement: Supplemental Information 1 [file peerj-cs-08-993-s001.zip › dataset files/Dataset/129.jpg]

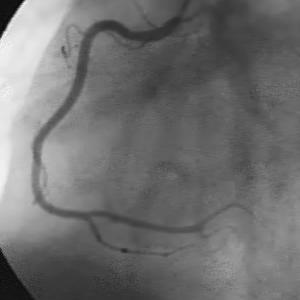

Supplement: Supplemental Information 1 [file peerj-cs-08-993-s001.zip › dataset files/Dataset/13.jpg]

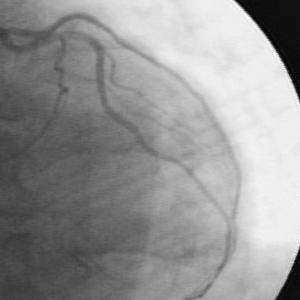

Supplement: Supplemental Information 1 [file peerj-cs-08-993-s001.zip › dataset files/Dataset/130.jpg]

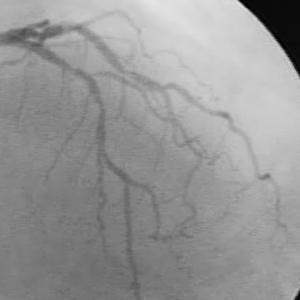

Supplement: Supplemental Information 1 [file peerj-cs-08-993-s001.zip › dataset files/Dataset/131.jpg]

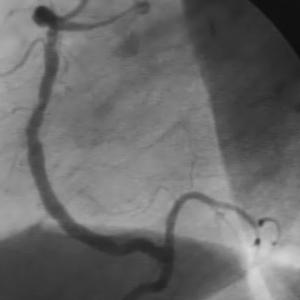

Supplement: Supplemental Information 1 [file peerj-cs-08-993-s001.zip › dataset files/Dataset/132.jpg]

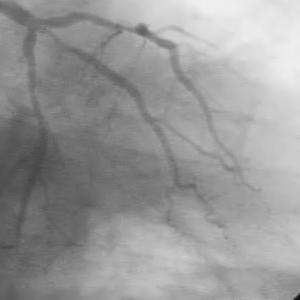

Supplement: Supplemental Information 1 [file peerj-cs-08-993-s001.zip › dataset files/Dataset/133.jpg]

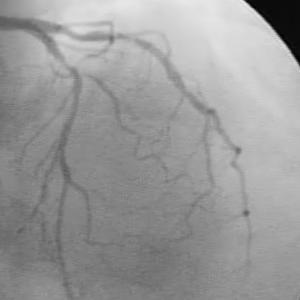

Supplement: Supplemental Information 1 [file peerj-cs-08-993-s001.zip › dataset files/Dataset/134.jpg]

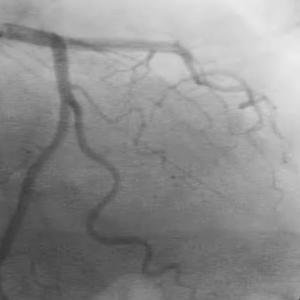

Supplement: Supplemental Information 1 [file peerj-cs-08-993-s001.zip › dataset files/Dataset/14.jpg]

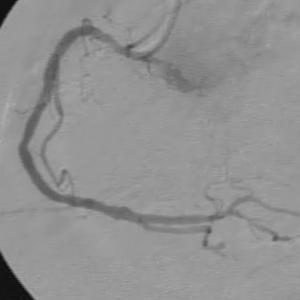

Supplement: Supplemental Information 1 [file peerj-cs-08-993-s001.zip › dataset files/Dataset/15.jpg]

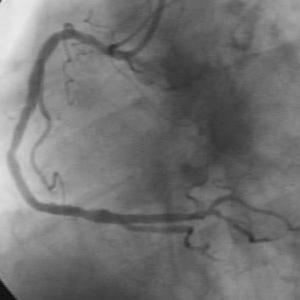

Supplement: Supplemental Information 1 [file peerj-cs-08-993-s001.zip › dataset files/Dataset/16.jpg]

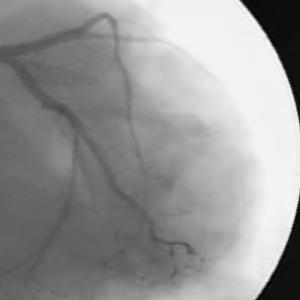

Supplement: Supplemental Information 1 [file peerj-cs-08-993-s001.zip › dataset files/Dataset/17.jpg]

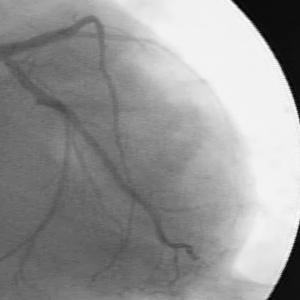

Supplement: Supplemental Information 1 [file peerj-cs-08-993-s001.zip › dataset files/Dataset/18.jpg]

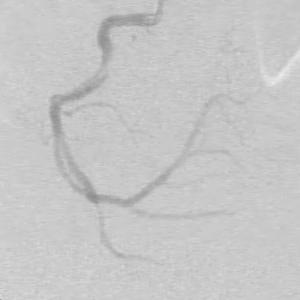

Supplement: Supplemental Information 1 [file peerj-cs-08-993-s001.zip › dataset files/Dataset/19.jpg]

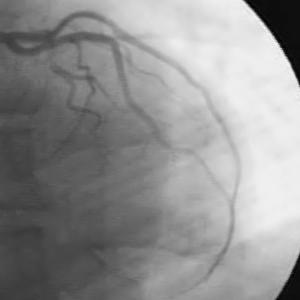

Supplement: Supplemental Information 1 [file peerj-cs-08-993-s001.zip › dataset files/Dataset/2.jpg]

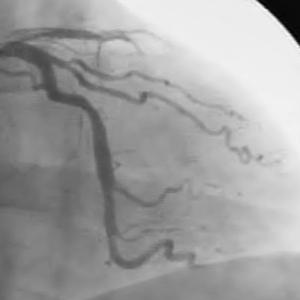

Supplement: Supplemental Information 1 [file peerj-cs-08-993-s001.zip › dataset files/Dataset/20.jpg]

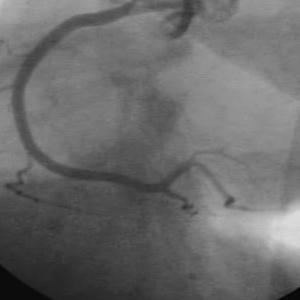

Supplement: Supplemental Information 1 [file peerj-cs-08-993-s001.zip › dataset files/Dataset/21.jpg]

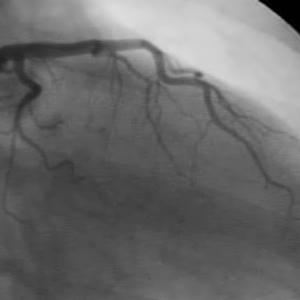

Supplement: Supplemental Information 1 [file peerj-cs-08-993-s001.zip › dataset files/Dataset/22.jpg]

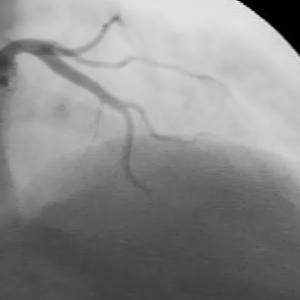

Supplement: Supplemental Information 1 [file peerj-cs-08-993-s001.zip › dataset files/Dataset/23.jpg]

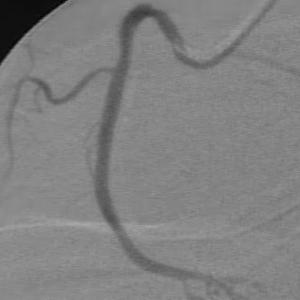

Supplement: Supplemental Information 1 [file peerj-cs-08-993-s001.zip › dataset files/Dataset/24.jpg]

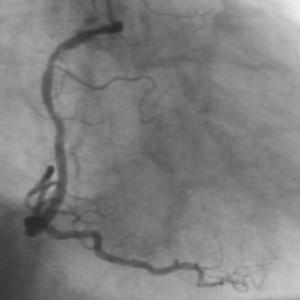

Supplement: Supplemental Information 1 [file peerj-cs-08-993-s001.zip › dataset files/Dataset/25.jpg]

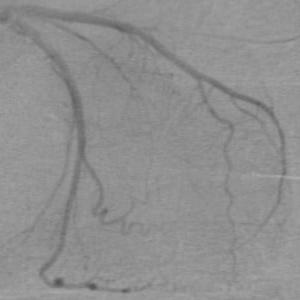

Supplement: Supplemental Information 1 [file peerj-cs-08-993-s001.zip › dataset files/Dataset/26.jpg]

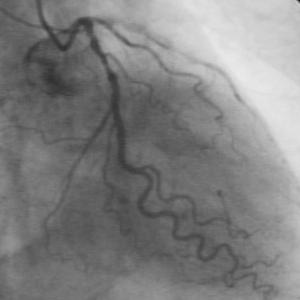

Supplement: Supplemental Information 1 [file peerj-cs-08-993-s001.zip › dataset files/Dataset/27.jpg]

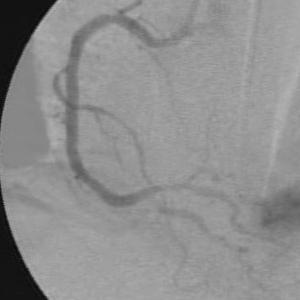

Supplement: Supplemental Information 1 [file peerj-cs-08-993-s001.zip › dataset files/Dataset/28.jpg]

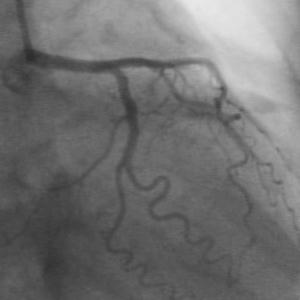

Supplement: Supplemental Information 1 [file peerj-cs-08-993-s001.zip › dataset files/Dataset/29.jpg]

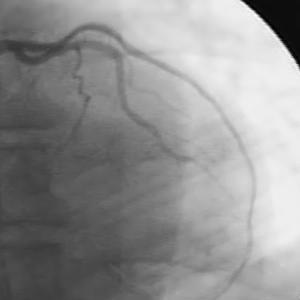

Supplement: Supplemental Information 1 [file peerj-cs-08-993-s001.zip › dataset files/Dataset/3.jpg]

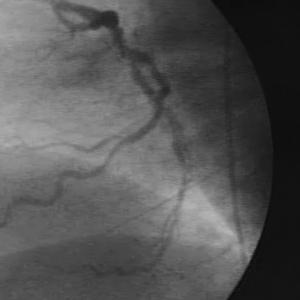

Supplement: Supplemental Information 1 [file peerj-cs-08-993-s001.zip › dataset files/Dataset/30.jpg]

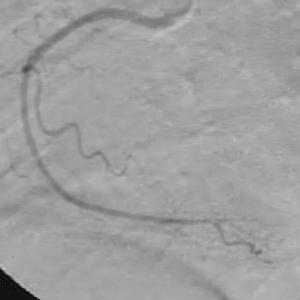

Supplement: Supplemental Information 1 [file peerj-cs-08-993-s001.zip › dataset files/Dataset/31.jpg]

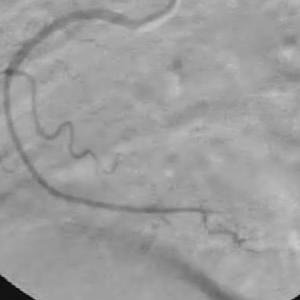

Supplement: Supplemental Information 1 [file peerj-cs-08-993-s001.zip › dataset files/Dataset/32.jpg]

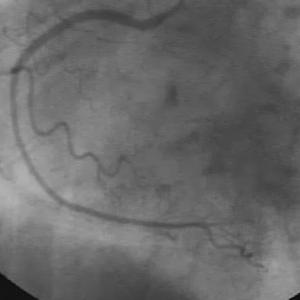

Supplement: Supplemental Information 1 [file peerj-cs-08-993-s001.zip › dataset files/Dataset/33.jpg]

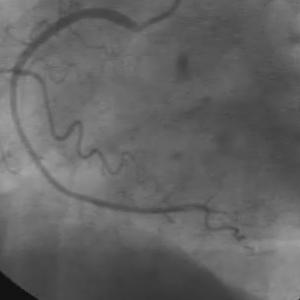

Supplement: Supplemental Information 1 [file peerj-cs-08-993-s001.zip › dataset files/Dataset/34.jpg]

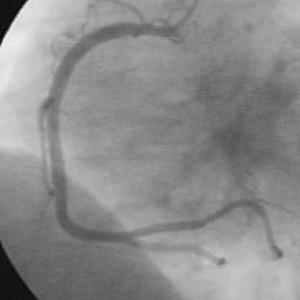

Supplement: Supplemental Information 1 [file peerj-cs-08-993-s001.zip › dataset files/Dataset/35.jpg]

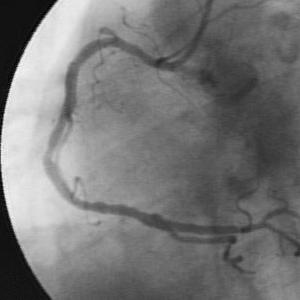

Supplement: Supplemental Information 1 [file peerj-cs-08-993-s001.zip › dataset files/Dataset/36.jpg]

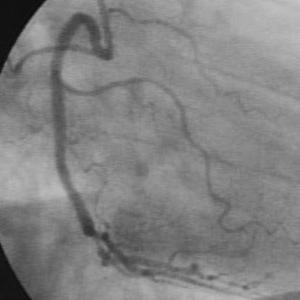

Supplement: Supplemental Information 1 [file peerj-cs-08-993-s001.zip › dataset files/Dataset/37.jpg]

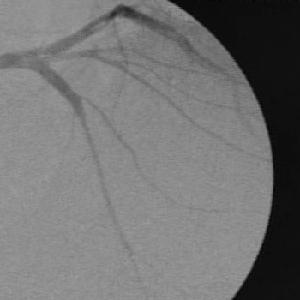

Supplement: Supplemental Information 1 [file peerj-cs-08-993-s001.zip › dataset files/Dataset/38.jpg]

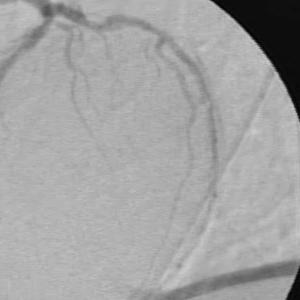

Supplement: Supplemental Information 1 [file peerj-cs-08-993-s001.zip › dataset files/Dataset/39.jpg]

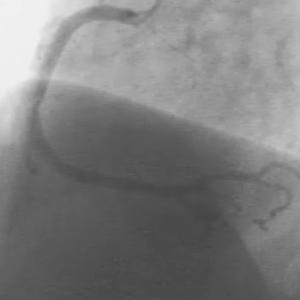

Supplement: Supplemental Information 1 [file peerj-cs-08-993-s001.zip › dataset files/Dataset/4.jpg]

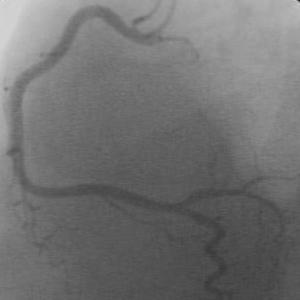

Supplement: Supplemental Information 1 [file peerj-cs-08-993-s001.zip › dataset files/Dataset/40.jpg]

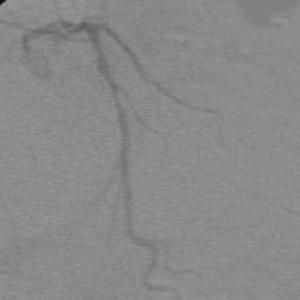

Supplement: Supplemental Information 1 [file peerj-cs-08-993-s001.zip › dataset files/Dataset/41.jpg]

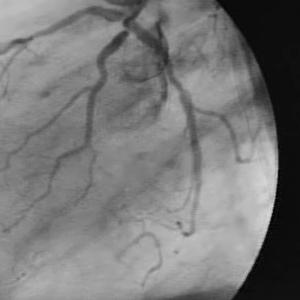

Supplement: Supplemental Information 1 [file peerj-cs-08-993-s001.zip › dataset files/Dataset/42.jpg]

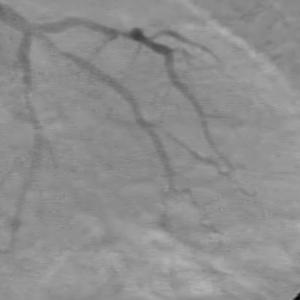

Supplement: Supplemental Information 1 [file peerj-cs-08-993-s001.zip › dataset files/Dataset/43.jpg]

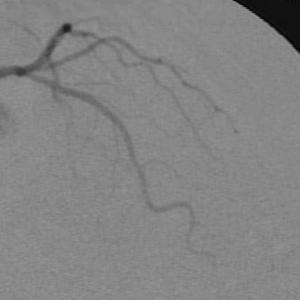

Supplement: Supplemental Information 1 [file peerj-cs-08-993-s001.zip › dataset files/Dataset/44.jpg]

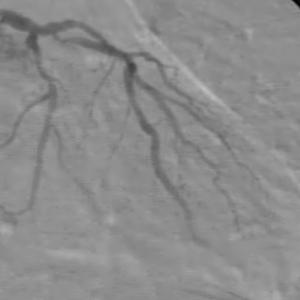

Supplement: Supplemental Information 1 [file peerj-cs-08-993-s001.zip › dataset files/Dataset/45.jpg]

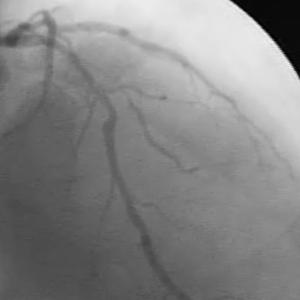

Supplement: Supplemental Information 1 [file peerj-cs-08-993-s001.zip › dataset files/Dataset/46.jpg]

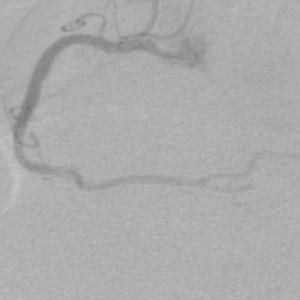

Supplement: Supplemental Information 1 [file peerj-cs-08-993-s001.zip › dataset files/Dataset/47.jpg]

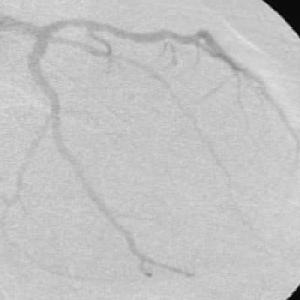

Supplement: Supplemental Information 1 [file peerj-cs-08-993-s001.zip › dataset files/Dataset/48.jpg]

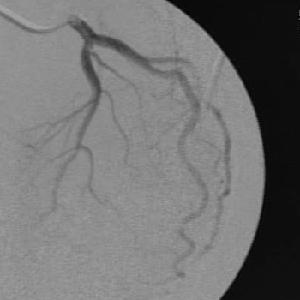

Supplement: Supplemental Information 1 [file peerj-cs-08-993-s001.zip › dataset files/Dataset/49.jpg]

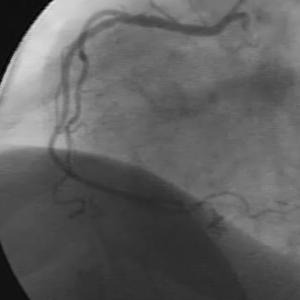

Supplement: Supplemental Information 1 [file peerj-cs-08-993-s001.zip › dataset files/Dataset/5.jpg]

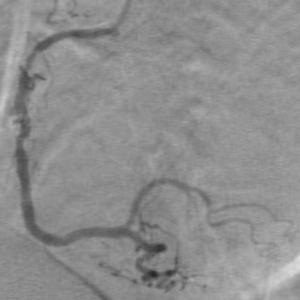

Supplement: Supplemental Information 1 [file peerj-cs-08-993-s001.zip › dataset files/Dataset/50.jpg]

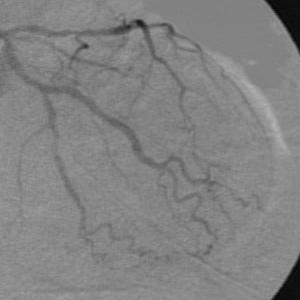

Supplement: Supplemental Information 1 [file peerj-cs-08-993-s001.zip › dataset files/Dataset/51.jpg]

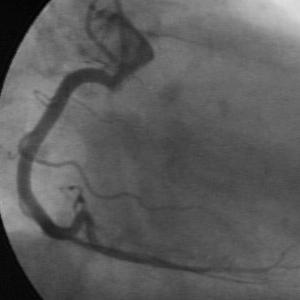

Supplement: Supplemental Information 1 [file peerj-cs-08-993-s001.zip › dataset files/Dataset/52.jpg]

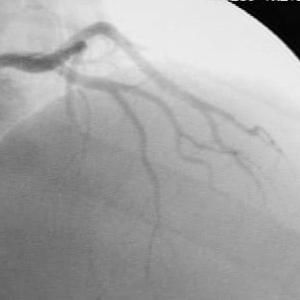

Supplement: Supplemental Information 1 [file peerj-cs-08-993-s001.zip › dataset files/Dataset/53.jpg]

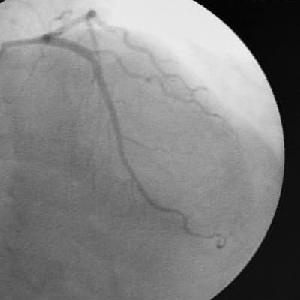

Supplement: Supplemental Information 1 [file peerj-cs-08-993-s001.zip › dataset files/Dataset/54.jpg]

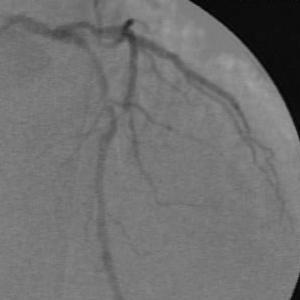

Supplement: Supplemental Information 1 [file peerj-cs-08-993-s001.zip › dataset files/Dataset/55.jpg]

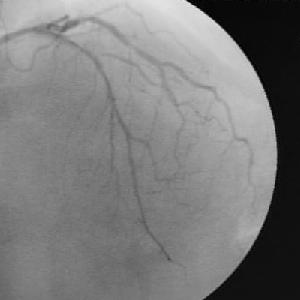

Supplement: Supplemental Information 1 [file peerj-cs-08-993-s001.zip › dataset files/Dataset/56.jpg]

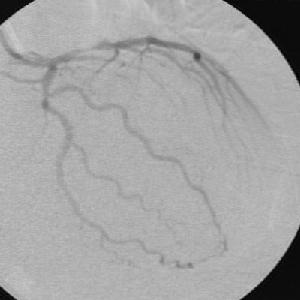

Supplement: Supplemental Information 1 [file peerj-cs-08-993-s001.zip › dataset files/Dataset/57.jpg]

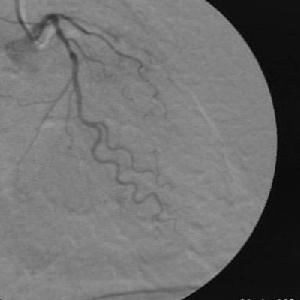

Supplement: Supplemental Information 1 [file peerj-cs-08-993-s001.zip › dataset files/Dataset/58.jpg]

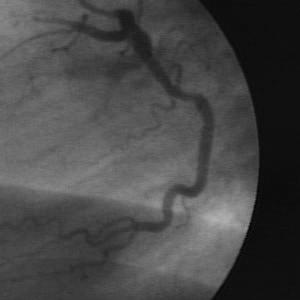

Supplement: Supplemental Information 1 [file peerj-cs-08-993-s001.zip › dataset files/Dataset/59.jpg]

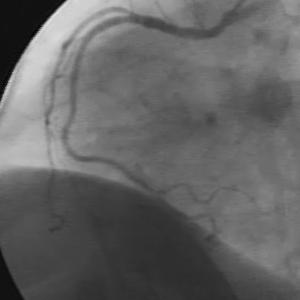

Supplement: Supplemental Information 1 [file peerj-cs-08-993-s001.zip › dataset files/Dataset/6.jpg]

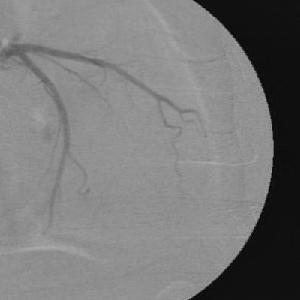

Supplement: Supplemental Information 1 [file peerj-cs-08-993-s001.zip › dataset files/Dataset/60.jpg]

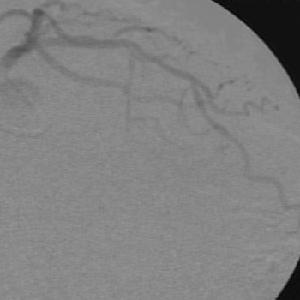

Supplement: Supplemental Information 1 [file peerj-cs-08-993-s001.zip › dataset files/Dataset/61.jpg]

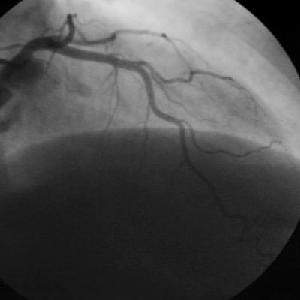

Supplement: Supplemental Information 1 [file peerj-cs-08-993-s001.zip › dataset files/Dataset/62.jpg]

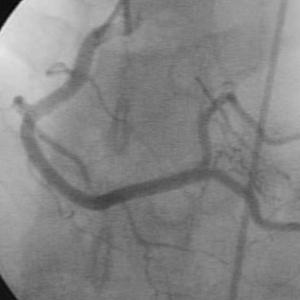

Supplement: Supplemental Information 1 [file peerj-cs-08-993-s001.zip › dataset files/Dataset/63.jpg]

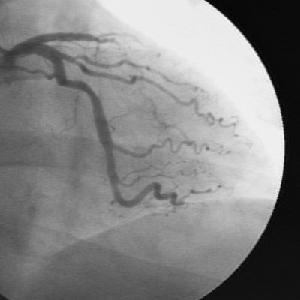

Supplement: Supplemental Information 1 [file peerj-cs-08-993-s001.zip › dataset files/Dataset/64.jpg]

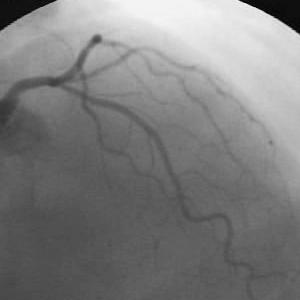

Supplement: Supplemental Information 1 [file peerj-cs-08-993-s001.zip › dataset files/Dataset/65.jpg]

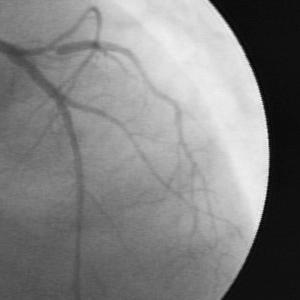

Supplement: Supplemental Information 1 [file peerj-cs-08-993-s001.zip › dataset files/Dataset/66.jpg]

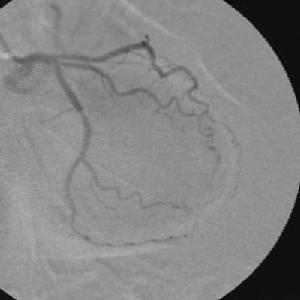

Supplement: Supplemental Information 1 [file peerj-cs-08-993-s001.zip › dataset files/Dataset/67.jpg]

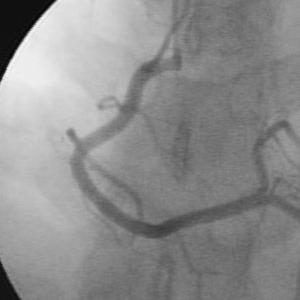

Supplement: Supplemental Information 1 [file peerj-cs-08-993-s001.zip › dataset files/Dataset/68.jpg]
